# Genetically predicted telomere length is associated with clonal somatic copy number alterations in peripheral leukocytes

**DOI:** 10.1371/journal.pgen.1009078

**Published:** 2020-10-22

**Authors:** Derek W. Brown, Shu-Hong Lin, Po-Ru Loh, Stephen J. Chanock, Sharon A. Savage, Mitchell J. Machiela

**Affiliations:** 1 Division of Cancer Epidemiology and Genetics, National Cancer Institute, Rockville, MD, United States of America; 2 Cancer Prevention Fellowship Program, Division of Cancer Prevention, National Cancer Institute, Rockville, MD, United States of America; 3 Division of Genetics, Department of Medicine, Brigham and Women’s Hospital and Harvard Medical School, Boston, MA, United States of America; 4 Program in Medical and Population Genetics, Broad Institute of MIT and Harvard, Cambridge, MA, United States of America; University of Chicago, UNITED STATES

## Abstract

Telomeres are DNA-protein structures at the ends of chromosomes essential in maintaining chromosomal stability. Observational studies have identified associations between telomeres and elevated cancer risk, including hematologic malignancies; but biologic mechanisms relating telomere length to cancer etiology remain unclear. Our study sought to better understand the relationship between telomere length and cancer risk by evaluating genetically-predicted telomere length (gTL) in relation to the presence of clonal somatic copy number alterations (SCNAs) in peripheral blood leukocytes. Genotyping array data were acquired from 431,507 participants in the UK Biobank and used to detect SCNAs from intensity information and infer telomere length using a polygenic risk score (PRS) of variants previously associated with leukocyte telomere length. In total, 15,236 (3.5%) of individuals had a detectable clonal SCNA on an autosomal chromosome. Overall, higher gTL value was positively associated with the presence of an autosomal SCNA (OR = 1.07, 95% CI = 1.05–1.09, P = 1.61×10^−15^). There was high consistency in effect estimates across strata of chromosomal event location (*e*.*g*., telomeric ends, interstitial or whole chromosome event; P_het_ = 0.37) and strata of copy number state (*e*.*g*., gain, loss, or neutral events; P_het_ = 0.05). Higher gTL value was associated with a greater cellular fraction of clones carrying autosomal SCNAs (β = 0.004, 95% CI = 0.002–0.007, P = 6.61×10^−4^). Our population-based examination of gTL and SCNAs suggests inherited components of telomere length do not preferentially impact autosomal SCNA event location or copy number status, but rather likely influence cellular replicative potential.

## Author summary

Telomeres lie at the ends of chromosomes and protect from damage and chromosomal fusions. Recent studies have identified relationships between telomere length and cancer risk; however, exactly how telomere length impacts cancer risk is unknown. We investigated potential associations between telomere length and somatic copy number alterations (SCNAs), as SCNAs are associated with cancer risk and may be responsible for some of the observed association between telomere length and cancer. We analyzed blood-derived DNA from over 430,000 participants in a large UK based study and identified an association between elevated telomere length (as inferred from genetic variants) and increased frequency of SCNAs. We did not find any difference in the relationship between telomere length and SCNAs based on specific regions of chromosomes impacted or whether chromosomes were lost or gained. However, we did note that longer predicted telomere length is associated with higher proportions of cells containing a SCNA. Although further studies are needed, our results provide new evidence indicating SCNAs may be a potential mechanism by which telomere length could impact cancer risk.

## Introduction

Telomeres consist of hexanucleotide DNA repeats and a protein structure at chromosome ends that protect genetic information by maintaining chromosomal stability during cellular division [1,2]. Each time a cell divides, small amounts of telomeric DNA are lost due to DNA polymerase’s inability to fully extend 3′ DNA ends [1]. Consequently, telomeres shorten with each cell division and can be a marker of cellular aging [1,3,4]. Telomere attrition over time results in critically short telomere lengths and leads naturally to cellular senescence and/or apoptosis in normal cells; cancer cells can bypass this process through upregulation of telomerase as well as inactivation of TP53 or RB or both [5,6]. In contrast, increased telomere length may promote tumorigenesis by allowing cells to continue to divide despite accumulation of mutations and genomic instability [5,7–9]. Epidemiological studies have found genetically- predicted telomere length (gTL) to be associated with increased risk of some hematologic malignancies (*e*.*g*. chronic lymphocytic leukemia, small lymphocytic lymphoma) [10] and solid tumors [2,11–13] but the biologic mechanisms connecting telomere length with cancer etiology remain unclear.

Somatic copy number alterations (SCNAs) are the presence of two or more genetically different cell populations in an individual [14]. Clonal expansion of cells harboring these SCNAs (*i*.*e*., non-inherited mutations in chromosome copy number resulting in genomic deletions or amplifications as well as copy neutral loss of heterozygosity) results in heterogeneous cellular populations with genetic mosaicism [14]. SCNAs in peripheral blood leukocytes have been robustly associated with increasing age, with approximately 10–20% of individuals acquiring a detectable autosomal SCNA by age 80 [15,16]. Clonal SCNAs in leukocytes have been associated with increased risk of hematopoietic cancers, such as leukemia, lymphoma, and multiple myeloma [17–20], suggesting that SCNAs may be representative of some sort of underlying chromosomal instability. SCNAs may be related to telomere length, as a representation of overall chromosome degradation or a manifestation of cellular replicative potential, and further serve as one of many potential mechanisms linking inherited telomere length to elevated cancer risk. Previous studies also suggest that the telomerase reverse transcriptase (*TERT*) gene is associated with both clonal hematopoiesis and autosomal mosaic events, further supporting a potential association between telomere length and clonal SCNAs [17,21,22].

To our knowledge, no prior study has systematically evaluated the relationship between telomere length and SCNAs, though studies on each measure separately have implicated these measures as risk factors for chronic diseases (*e*.*g*., cancer, diabetes) [2,14]. This study aimed to use existing UK Biobank genotyping array data to evaluate the association between gTL and clonal SCNAs.

## Results

A total of 431,507 individuals with complete genotyping data were included in this analysis. Mean gTL was similar between males and females (P = 0.6360), but differed by age quartiles, smoking status, and by self-report ethnicity (P<2x10^-16^; **Table 1**). The differences observed in gTL by age quartile and smoking status were no longer significant when adjusted for self-reported ethnicity (P = 0.0800 and P = 0.4295, respectively; **S1** and **S2 Tables**) as both age and smoking vary by ethnicity, which is an important determinant of gTL. Participant demographic characteristics by autosomal SCNA status are described in **Table 2**. Overall, 15,236 (3.5%) participants had at least one detectable clonal SCNA on an autosomal chromosome. Compared to individuals without autosomal SCNAs, those with autosomal SCNAs were more likely to be male, tended to be older, more likely to be former or current smokers, and had a higher proportion of European genetic ancestry (P<2x10^-16^). Additionally, subjects with autosomal SCNAs had on average significantly higher gTL value compared to those without autosomal SCNAs (P = 3.54×10^−9^).

**Table 1:** Genetically-predicted telomere length by UK Biobank population characteristics.

Characteristic	Mean (SD)	p-value
Sex		0.636
Male	0.00 (1.00)	
Female	0.00 (1.00)	
Age Quartile		<2x10^-16^
≤50	0.03 (1.01)	
51–58	0.00 (1.00)	
59–63	-0.01 (1.00)	
≥64	-0.02 (0.99)	
Smoking Status		<2x10^-16^
Never	0.01 (1.00)	
Former	-0.02 (0.99)	
Current	0.01 (1.00)	
Missing	0.06 (1.05)	
Ethnicity		<2x10^-16^
White	-0.03 (0.99)	
Black	1.07 (0.87)	
Asian	0.37 (1.00)	
Other	0.36 (1.04)	
Missing	0.15 (1.05)	

**Table 2:** UK Biobank population characteristics by autosomal SCNAs status.

	Autosomal Mosaicism		
Characteristic	Yes	No	p-value
N	15,263	416,244	-
Sex, N (%)			<2x10^-16^
Male	7,618 (49.90)	189,722 (45.60)	
Female	7,645 (50.10)	226,522 (54.40)	
Age, Mean (SD)	59.48 (7.39)	56.43 (8.10)	<2x10^-16^
Age Quartile, N (%)			<2x10^-16^
≤50	2,234 (14.64)	111,080 (26.69)	
51–58	3,319 (21.75)	110,820 (26.62)	
59–63	4,215 (27.62)	98,094 (23.57)	
≥64	5,495 (36.00)	96,250 (23.12)	
Smoking Status, N (%)			<2x10^-16^
Never	7,675 (50.30)	227,346 (54.60)	
Former	5,745 (37.70)	142,028 (34.10)	
Current	1,691 (11.10)	42,976 (10.30)	
Missing	150 (1.00)	3,894 (0.90)	
Ethnicity, N (%)			<2x10^-16^
White	14,720 (96.40)	39,1942 (94.20)	
Black	131 (0.90)	6,703 (1.60)	
Asian	204 (1.30)	9,414 (2.30)	
Other	153 (1.00)	6,367 (1.50)	
Missing	55 (0.40)	1,818 (0.40)	
Genetic Ancestry, Mean (SD)			<2x10^-16^
European	0.94 (0.10)	0.93 (0.14)	
African	0.05 (0.08)	0.06 (0.11)	
Asian	0.01 (0.07)	0.01 (0.09)	
gTL, Mean (SD)	0.05 (1.00)	0.00 (1.00)	3.54x10^-9^

gTL = genetically-predicted telomere length

**Fig 1:**
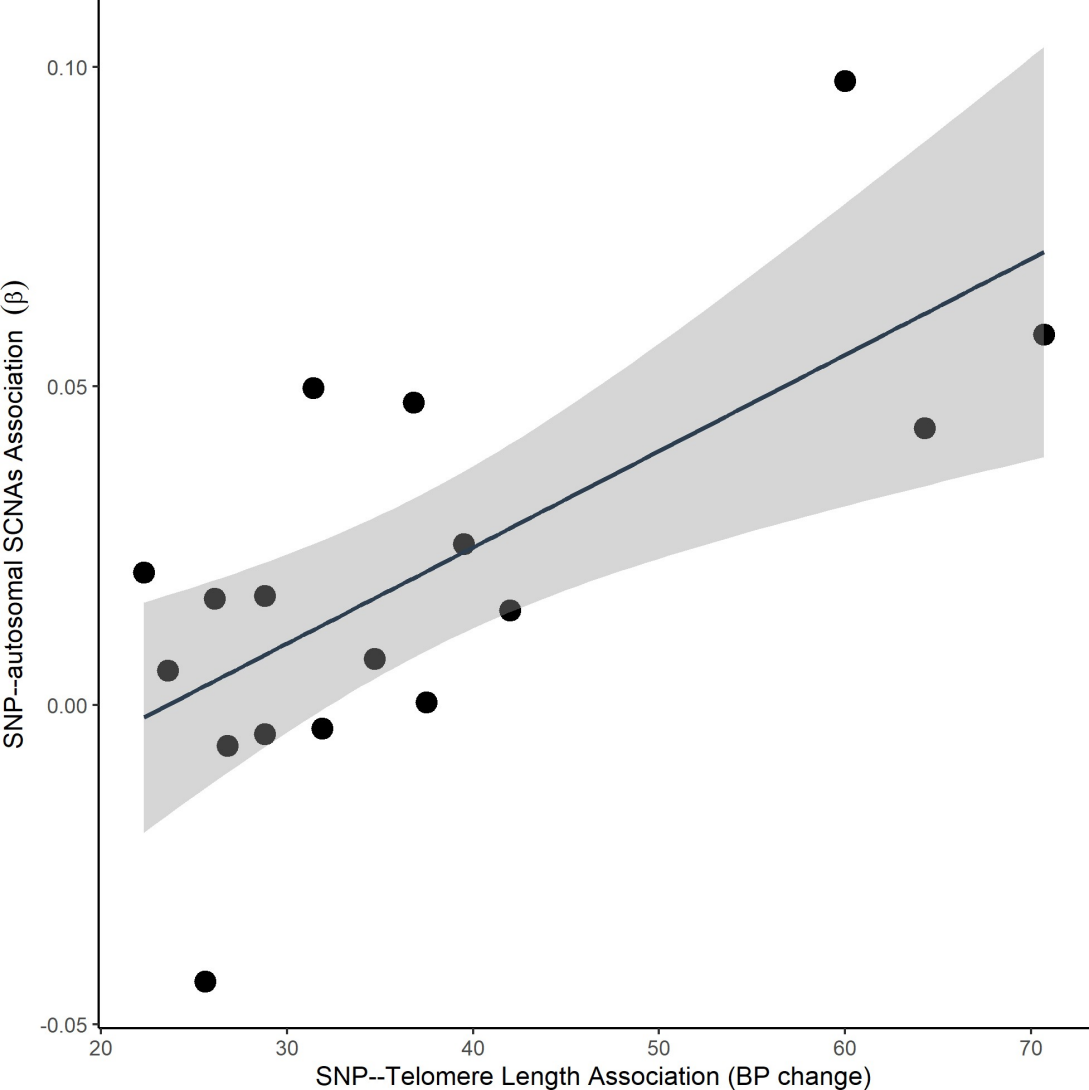
The effect of each variant on genetically-predicted telomere length and autosomal SCNAs. Estimates for the single nucleotide polymorphism (SNP)—telomere and SNP—autosomal SCNAs associations are presented in **S4 Table**. Only variants with minor allele frequency greater than 5% are included.

Univariable and multivariable analyses for the association between gTL and the presence of autosomal SCNAs are given in **Table 3**. Overall, results from multivariable analyses showed a positive association between having an autosomal SCNA and gTL value (OR = 1.07, 95% CI = 1.05–1.09, P = 1.61×10^−15^), suggesting an elevated risk of autosomal SCNAs in individuals with higher gTL. Sensitivity analyses adjusting for the first 10 principal components, rather than inferred ancestry proportions, produced nearly identical association results (OR = 1.07, 95% CI = 1.05–1.09, P = 1.18×10^−15^). Associations were consistent when stratified by sex (Male: OR = 1.07, 95% CI = 1.05–1.10, P = 9.18×10^−9^; Female: OR = 1.07, 95% CI = 1.04–1.09, P = 3.62×10^−8^, P_het_ = 0.8588), age (≥64 years: OR = 1.08, 95% CI = 1.05–1.11, P = 5.26×10^−8^; ≤50: OR = 1.03, 95% CI = 0.98–1.07, P = 0.2219, P_het_ = 0.2207), and self-reported ethnicity (White: OR = 1.07, 95% CI = 1.06–1.09, P<2×10^−16^, P_het_ = 0.1484; **S3 Table**). Similar positive associations were also observed between specific individual telomere length-associated SNPs and the odds of autosomal SCNAs (**Fig 1** and **S4 Table**). Of the 20 telomere length-associated variants included in our derived PRS, six variants (rs2293607, rs7705526, rs2488002, rs78517833, rs11846938, and rs28616016) displayed evidence for an individual association with SCNA (*p* < 0.05). For all the telomere length-related variants associated with SCNAs, the allele related to longer telomere length was associated with an increased odds of developing SCNAs. Mendelian randomization (MR) analyses further indicated a directionality between telomere length-associated variants and SCNA (**S5 Table, S6 Table** and S3 Fig). After removing detected heterogeneous variants, the intercept from MR-Egger regression was non-significant (P = 0.2375), suggesting no significant evidence for directional pleiotropy on the remaining variants. The maximum-likelihood based method predicted an increasing effect between the telomere length genetic instrument and the odds of SCNAs (OR = 1.72, 95% CI = 1.48–2.00, P = 7.51×10^−13^; **S6 Table**). Likewise, similar results were viewed across a variety of MR methods (**S6 Table** and S3 Fig).

**Table 3:** Association between genetically-predicted telomere length and autosomal SCNAs by event type and copy number change.

	Univariable Model		Multivariable Model		
	OR (95% CI)	p-value	OR (95% CI)	p-value	p-value_het_^a^
Overall	1.05 (1.033–1.067)	3.61x10^-9^	1.07 (1.052–1.087)	1.61x10^-15^	
Event Type					0.3707
Telomeric	1.05 (1.027–1.067)	2.19x10^-6^	1.07 (1.045–1.086)	1.13x10^-10^	
Interstitial	1.08 (1.042–1.129)	6.47x10^-5^	1.10 (1.057–1.146)	3.51x10^-6^	
Whole	1.05 (1.005–1.088)	0.0262	1.07 (1.028–1.114)	0.0009	
Copy Number Change					0.0502
Gain	1.04 (0.995–1.089)	0.0788	1.06 (1.009–1.106)	0.0191	
Loss	1.10 (1.064–1.147)	1.99x10^-7^	1.13 (1.085–1.171)	8.46x10^-10^	
Neutral	1.05 (1.026–1.075)	3.92x10^-5^	1.07 (1.044–1.095)	3.31x10^-8^	
Undetermined	1.04 (1.006–1.067)	0.0185	1.06 (1.025–1.088)	3.51x10^-4^	

Multivariable models control for sex, age, age^2^, genetic ancestry, and detailed smoking status.

^a^Denotes test of heterogeneity.

Further analyses stratified by chromosomal event location (telomeric, interstitial or whole chromosome events) and copy number state (gain, loss, neutral, or undetermined events) also identified an association between autosomal SCNA status and higher gTL value regardless of SCNA chromosomal location or copy number state (**Table 3**). Tests for heterogeneity indicated no evidence for differences in associations by chromosomal event locations (P_het_ = 0.3707) or copy number states (P_het_ = 0.0502).

Analyses were also conducted to explore whether the proportion of cells impacted by autosomal SCNAs was associated with gTL. Multivariable results found a positive association such that as gTL increases, so does the proportion of cells with autosomal SCNAs (β = 0.004, 95% CI = 0.002–0.007, P = 6.61×10^−4^). Similarly, higher gTL value was associated with a greater expected number of autosomal SCNAs among participants with autosomal SCNAs (IRR = 1.07, 95% CI = 1.02–1.11, P = 1.94×10^−3^). The distribution of the total number of autosomal events for each participant is given in **S1 Fig**.

All analyses were repeated to additionally assess the association between gTL and SCNAs within the sex chromosomes (chromosome X and Y). In total, 12,200 (5.2%) female participants had a chromosome X SCNA, and 38,685 (19.6%) male participants had chromosome Y loss. Only chromosome Y loss was considered for our analyses, as the majority of chromosome Y SCNAs represent a loss. Overall, there was a positive association between gTL and chromosome X SCNAs (OR = 1.04, 95% CI = 1.03–1.06, P = 5.87×10^−6^; **S7 Table**). gTL was also positively associated with the proportion of cells affected with chromosome X SCNAs (β = 0.002, 95% CI = 0.0002–0.003, P = 2.35×10^−2^), but not the expected number of chromosome X SCNAs (IRR = 1.45, 95% CI = 0.89–2.35, P = 0.1300) as few women had more than 1 chromosome X event (N = 14). Conversely, overall analyses suggested gTL was negatively associated with mosaic loss of the Y chromosome (OR = 0.97, 95% CI = 0.96–0.99, P = 2.71×10^−5^). Subset analyses by mosaic fraction suggest low cell fraction events (LRR > -0.05; N = 25,338) are the predominant driver of this negative association between gTL and mosaic loss of the Y chromosome; however, analyses on a small set of individuals with greater degrees of mosaic loss of Y (LRR ≤ -0.40; N = 505) suggest a positive association may exist for higher cell fraction events (LRR ≤ -0.40: OR = 1.10, 95% CI = 1.01–1.21, P = 0.0340; **S8 Table**).

## Discussion

Our findings from this large observational study of 431,507 individuals suggest that gTL is associated with the presence of autosomal SCNAs in peripheral blood leukocytes. We observed consistent associations between gTL and autosomal SCNAs by strata of chromosomal event location and copy number state. We observed gTL was positively associated with both the autosomal SCNA cellular fraction and number of SCNA events. These results suggest telomere length has potentially less influence on chromosomal location or copy number status of SCNAs, but rather that longer telomeres could be associated with clonal expansion of SCNAs by increasing cellular replicative potential.

This study represents the first population-based study to assess the relationship between gTL and SCNAs. As measured telomere length was not available from the UK Biobank data, our study used genetic variants associated with leukocyte telomere length as a proxy for measured telomere length [23]. This genetic approach to estimate telomere length does not contain the biases generally attributable to measured telomere length (*e*.*g*., differences in DNA extraction or storage [24]). Several studies have demonstrated the power of this approach to identify associations between telomere length and a variety of outcomes [2,10–13,25,26]. Additionally, previous studies that have derived gTL using a PRS have incorporated variant weights from several different genome-wide association studies (GWAS) [2,10,11], which may lead to uncomparable weight estimates due to study specific differences. Our study only utilized weights from a single large telomere length GWAS [23]. This ensures that all SNP weights are on the same scale, potentially leading to a more accurate estimate of gTL within our study.

SCNAs in the sex chromosomes were analyzed separately from the autosomes as mosaic events on the sex chromosomes vary by frequency and location on the sex chromosomes [19,27,28]. The positive associations found between gTL and autosomal SCNAs were also observed within chromosome X SCNAs. This replication further supports a relationship between gTL and SCNAs. Although an association between gTL and mosaicism was observed with chromosome Y SCNAs, the observed association was negative, potentially reflecting unique molecular drivers of somatic copy number alterations on the haploid male Y chromosome [29]. Further analyses of prior identified Y loss susceptibility variants around telomere-related genes (*e*.*g*., *RPN1*, *TERT*, *ATM*, *TCL1A*) [30–32] indicated these variants were in linkage disequilibrium and negatively associated with telomere length increasing variants [33], suggesting some component of telomere length may be an important contributor to mosaic Y loss even though the direction of association differs for mosaic Y loss. The telomere length-associated PRS used in our current analysis contains only currently discovered telomere length-associated variants, which likely represents only a small fraction of all genetic variants associated with telomere length and therefore may not capture all telomere length associations.

Our study is not without limitations. While the original GWAS results used to derive our PRS for gTL found 22 telomere length-associated variants [23], two of the variants, rs547680822 and rs4027719, were not found in the UK Biobank imputed data. rs547680822 has a reported alternate allele frequency of 0% within TOPMed European samples [23], so it is not surprising that the variant was not found within our data. Additionally, rs4027719 is an indel on chromosome 11 which was not included in the UK Biobank imputed genotype data. While our analysis is missing data on these two telomere length-associated variants, the resulting missingness is minimal, especially for the rs547680822 variant which is rare in Europeans, and is not anticipated to appreciably change analytic conclusions. Furthermore, the original TOPMed GWAS results did not include standard errors for the weights of the 22 telomere length-associated variants [23], which precluded rigorous MR tests to be conducted with our original panel of included variants. Instead, MR tests were conducted using available summary statistics from a telomere length GWAS performed in Europeans [34]. This alternative telomere length variant panel has considerable overlap with our original panel, but also includes a few novel loci. Results from MR analyses provide additional evidence for a directional relationship between the telomere length-associated variants and SCNAs.

Our analyses suggest a connection between longer gTL and SCNAs at the population level. Specifically, our analyses indicate cellular fraction of SCNA clones is the predominant driver of the association between gTL and SCNAs, indicating that inherited telomere length may be important for clonal expansion of hematopoietic stem cells harboring SCNAs. Additional studies are needed to further evaluate the association between telomere length and clonal SCNAs, particularly in relation to cancer risk. Prospective studies, which collect serial samples, may be useful in evaluating joint impacts of telomere length and clonal SCNAs over time and further help to explore possible biologic mechanisms that may lead to improved etiologic understanding, with potential relevance for cancer risk modeling and cancer prevention.

## Material and methods

### Data source

Existing data from the UK Biobank was utilized to investigate the association between leukocyte telomere length and SCNAs. Briefly, the UK Biobank is a large prospective study based in the United Kingdom which collected blood samples for genotyping as well as medical history and environmental exposures from study participants between 2006 and 2010 [35]. In total, approximately 500,000 participants were genotyped, and additional health outcomes data are available from linked UK national registries and hospital records. Each participant provided signed informed consent at enrollment. Blood samples were provided by participants after informed consent and sent to a central laboratory to be processed, aliquoted and cryopreserved. All research was performed in accordance with relevant regulations, and the UK Biobank study was approved by the National Information Governance Board for Health and Social Care and the National Health Service North West Multi-centre Research Ethics Committee. Data used in this analysis is available through application to the UK Biobank.

### Study variables

Participant characteristics were obtained from questionnaire results at the time of enrollment. Based on these results, a detailed 25-level smoking variable was created to give a complete overview of each participant’s lifetime smoking history which included information on smoking status, smoking duration, smoking intensity, time since quitting (for former smokers), and the type of tobacco smoked [29]. Genetic ancestry proportions were also inferred for each participant using SNPWEIGHTS which uses SNP weights computed from large reference panels to estimate genetic ancestry [36]. This approach to estimate ancestry has several advantages over reference-free approaches (e.g., principal component analysis), namely no dependency on sample size and utilization with related individuals [36,37]. The percentage of African, Asian, and European ancestry was computed for each participant, with European ancestry serving as the reference category in analyses. We also performed principal component analysis with SMARTPCA [38,39] on all included UK Biobank participants using the ancestry informative SNP panel described by Yu et al. [40] to calculate the first 10 principal components for a sensitivity analysis in place of genetic ancestry proportions.

Measured leukocyte telomere length was not available within the UK Biobank data. Instead, for each study participant, leukocyte telomere length was estimated using a genetic profile estimated from a polygenic risk score (PRS) [2,11]. This telomere length-associated PRS explains approximately 1.5% of the variation in leukocyte telomere length and contains 22 germline genetic variants that were identified to be associated with leukocyte telomere length within a GWAS of TOPMed whole genome sequencing data (**Table 4**) [23]. The telomere length-associated PRS was calculated for the 22-telomere length-associated variants as:

$$PRS_{i}=\sum_{j=1}^{22}w_{j}x_{ij}$$ (1)

where *PRS_i_* is the risk score for individual *i, w_j_* is the weight assigned to each telomere length-associated variant given as the change in the number of estimated base pairs per telomere length-increasing allele, and *x_ij_* is the number of individual-specific telomere length-increasing alleles for the *j^th^* telomere length-associated variant. This PRS was further standardized to have mean 0 and standard deviation of 1 and used in analyses as a proxy for telomere length, where higher standardized PRS value indicates longer gTL.

**Table 4:** Significant telomere length-associated germline genetic variants identified from TOPMed analysis.

Nearby gene	CHR	Position (hg37)	SNP^a^	Ref	Alt	UKBB AAF	TOPMed AAF	TOPMed BP
ACYP2	2	54495222	rs7579722	G	C	0.15	0.15	37.5
RPN1	3	128422176	rs60092972	A	T	0.31	0.32	23.6
TERC	3	169482335	rs2293607	C	T	0.76	0.76	70.7
NAF1	4	164048199	rs4691895	G	C	0.78	0.76	39.5
TERT	5	1285974	rs7705526	C	A	0.32	0.33	60
POT1	7	124494861	rs10246424	G	A	0.73	0.72	28.8
OPRK1	8	54434760	rs188891454	C	T	1.00	1.00	234
LINC01592	8	70243701	rs144510686	T	G	1.00	1.00	382.3
TERF1	8	73950559	rs12679652	A	G	0.32	0.32	28.8
SH3PXD2A	10	105679341	rs2488002	T	C	0.17	0.17	64.3
TINF2	14	24711798	rs28372734	C	G	0.00	0.00	152.1
DCAF4	14	73432100	rs78517833	A	T	0.10	0.10	36.8
TCL1A	14	96180685	rs11846938	T	G	0.24	0.24	25.6
TERF2	16	69391714	rs9925619	C	G	0.29	0.28	26.8
RFWD3	16	74676964	rs28616016	C	T	0.58	0.58	31.4
ZNF676	19	22424997	rs281173	G	A	0.62	0.63	22.3
SAMHD1	20	35578680	rs4810362	G	A	0.85	0.85	34.7
LINC01429	20	50453984	rs6091385	C	T	0.11	0.12	31.9
RTEL1	20	62336258	rs6062497	T	C	0.33	0.31	42
CHKB	22	51034870	rs131742	G	A	0.62	0.62	26.1

^a^Two SNPs were not identified in UKBB: rs547680822 (TOPMed AAF = 0.00), rs4027719 (TOPMed AAF = 0.43)

AAF = Alternate allele frequency

BP = Base pairs

Clonal SCNAs were detected in study participants using intensity values and haplotype information from SNP genotyping data obtained by hybridizing blood-derived DNA to SNP microarrays (Affymetrix UK BiLEVE Axiom and UK Biobank Axiom arrays) [17,41,42]. Specifically, estimates of log_2_ *R* ratio (LRR), B allele frequency (BAF) and genetic haplotype were used to detect large structural clonal SCNAs [17,42]. LRR provides a metric for assessing copy-number change (*e*.*g*., losses versus gains); whereas, BAF is a measure of allelic imbalance (*i*.*e*., deviations from Mendelian allelic fractions) [42]. SCNA calls within autosomal chromosomes and the Y chromosome were previously generated using a hidden Markov model-based approach that detects allelic imbalances in long-range phased haplotypes (analyzing only the pseudoautosomal region for the chromosome Y) [17,30,41]. We extended the previous analysis to the X chromosome, phasing all individuals together using Eagle2 as previously described [17,43] and then restricting the calling algorithm to females. To obtain improved estimates of mosaic fraction for Y chromosome events, LRR values were combined across the whole chromosome after removal of pseudoautosomal regions [29].

The copy number state of each clonal SCNA was further characterized as gain, loss, neutral, or undetermined based on LRR deviation [17]. Events with low cellular fraction have small deviations in LRR values and are difficult to classify into a distinct copy number state. These events were not called for copy number state and categorized as undetermined events. Clonal SCNAs were also categorized based on where in the chromosome the event occurred (*e*.*g*., telomeric ends, centromeric, interstitial, or whole chromosome event; **S2 Fig**). Chromosome size as well as centromeric positions were pulled from the UCSC Human Genome Browser [44]. Events were defined as follows: (1) events which only occurred around telomeric ends (±1 Mb from chromosome ends) were defined as telomere events, (2) events which crossed the centromere were defined as centromeric, (3) events that spanned an entire chromosome were defined as whole chromosome events, and (4) all other events were defined as interstitial.

### Statistical analysis

Demographic characteristics were first described by gTL and autosomal SCNA status (yes/no). Multivariable logistic regression models were then used to assess the association between genetically predicted telomere length and autosomal SCNA status. Further multivariable models categorized SCNA status by both chromosomal event location and copy number state. The association between the autosomal SCNA cellular fraction, given as the proportion of cells affected, and gTL was conducted using a multivariable linear model. The number of autosomal SCNAs, given as a count variable, were further analyzed with multivariable Poisson regression models. In addition to the performed gTL analyses with a PRS, a variety of MR analyses were conducted in order to combine telomere length-associated variants into a genetic instrument [45–47]. As standard errors were not available for the 22 included TOPMed telomere length-associated variants, other available variants and summary statistics were used to conduct MR analyses [34]. Heterogeneity among the included variants was investigated, and any variants with detected pleiotropic effects (i.e., false discovery rate < 0.2) were removed from subsequent MR analyses [48]. Multivariable analyses adjusted for sex, age, age-squared (age^2^), genetic ancestry, and detailed smoking status (25-level indicator variables). All statistical analyses were performed using a 64-bit build of R version 3.5.2 and two-sided significance levels were set at P < 0.05.

## Supporting information

S1 Fig The distribution of the total number of autosomal SCNAs per UK Biobank participant. Of those with autosomal SCNAs, most participants only had 1 or 2 events.
(DOCX)

S2 Fig Categorization of clonal SCNAs by chromosome event location (e.g., telomeric ends, centromeric, interstitial, or whole chromosome event). (DOCX)

S3 Fig Mendelian randomization estimates between telomere length-associated variants and autosomal somatic copy number alterations. Plot (a) contains all 20 telomere length-associated variants from Li et al. (2020); plot (b) removes pleiotropic variants as detailed in **S5 Table**.
(DOCX)

S1 Table UK Biobank population proportion breakdown by age and ethnicity. (DOCX)

S2 Table Genetically-predicted telomere length by age and ethnicity. (DOCX)

S3 Table Stratified associations between genetically-predicted telomere length and autosomal SCNAs. (DOCX)

S4 Table Associations of telomere length-associated variants from Taub et al. (2019) with autosomal SCNAs. (DOCX)

S5 Table Associations of telomere length-associated variants from Li et al. (2020) with autosomal SCNAs. (DOCX)

S6 Table Mendelian randomization results using variants and summary statistics from Li et al. (2020). (DOCX)

S7 Table Association between genetically-predicted telomere length and chromosome X SCNAs by event type and copy number change. (DOCX)

S8 Table Association between genetically-predicted telomere length and chromosome Y loss by log_2_ R ratio. (DOCX)

S1 Code Supporting Code. (ZIP)
