# Supplementary material for: Genetically predicted telomere length is associated with clonal somatic copy number alterations in peripheral leukocytes
Source: PLoS Genet. 2020 Oct 22;16(10):e1009078. doi: 10.1371/journal.pgen.1009078 (PMC7608979; doi:10.1371/journal.pgen.1009078)
Supplement: S1 Fig — Of those with autosomal SCNAs, most participants only had 1 or 2 events. (DOCX) [file pgen.1009078.s001.docx]

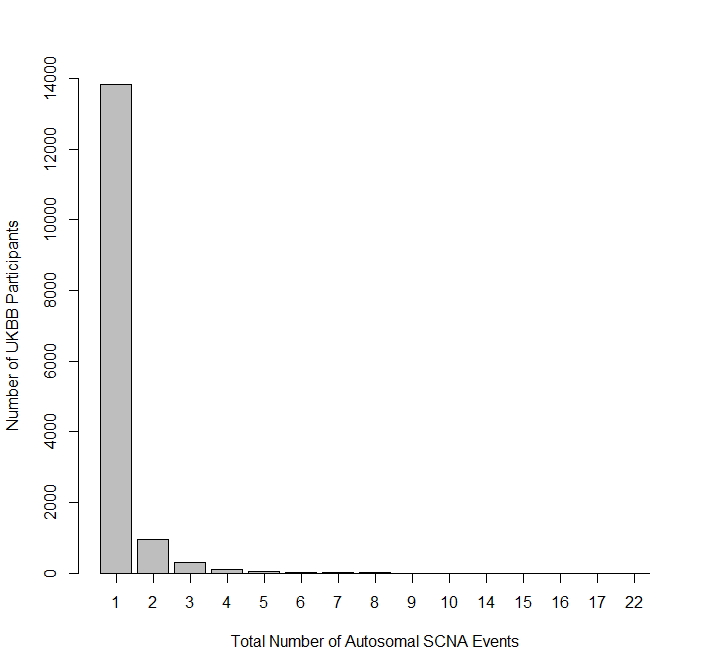


**S1 Fig**. The distribution of the total number of autosomal SCNAs per UK Biobank participant. Of those with autosomal SCNAs, most participants only had 1 or 2 events.
