# Supplementary material for: Genetically predicted telomere length is associated with clonal somatic copy number alterations in peripheral leukocytes
Source: PLoS Genet. 2020 Oct 22;16(10):e1009078. doi: 10.1371/journal.pgen.1009078 (PMC7608979; doi:10.1371/journal.pgen.1009078)
Supplement: S2 Fig — (DOCX) [file pgen.1009078.s002.docx]

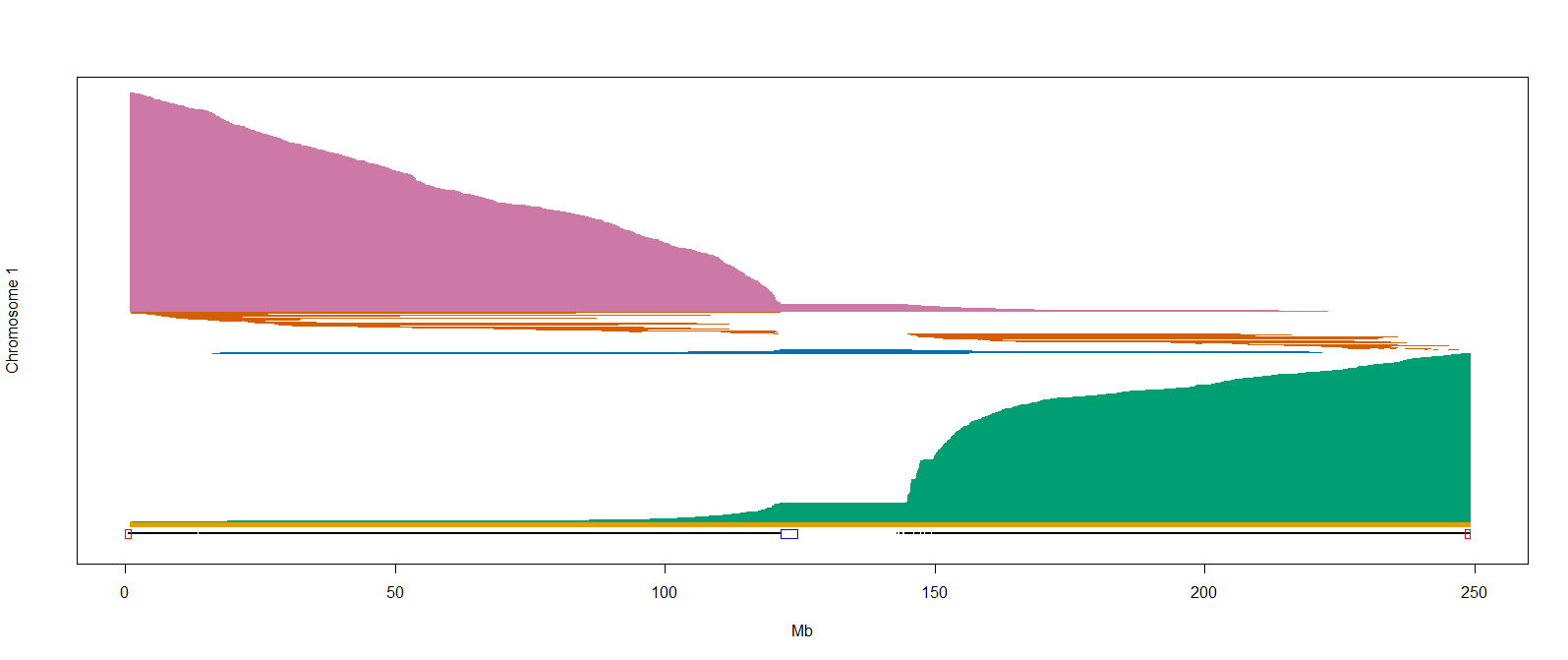


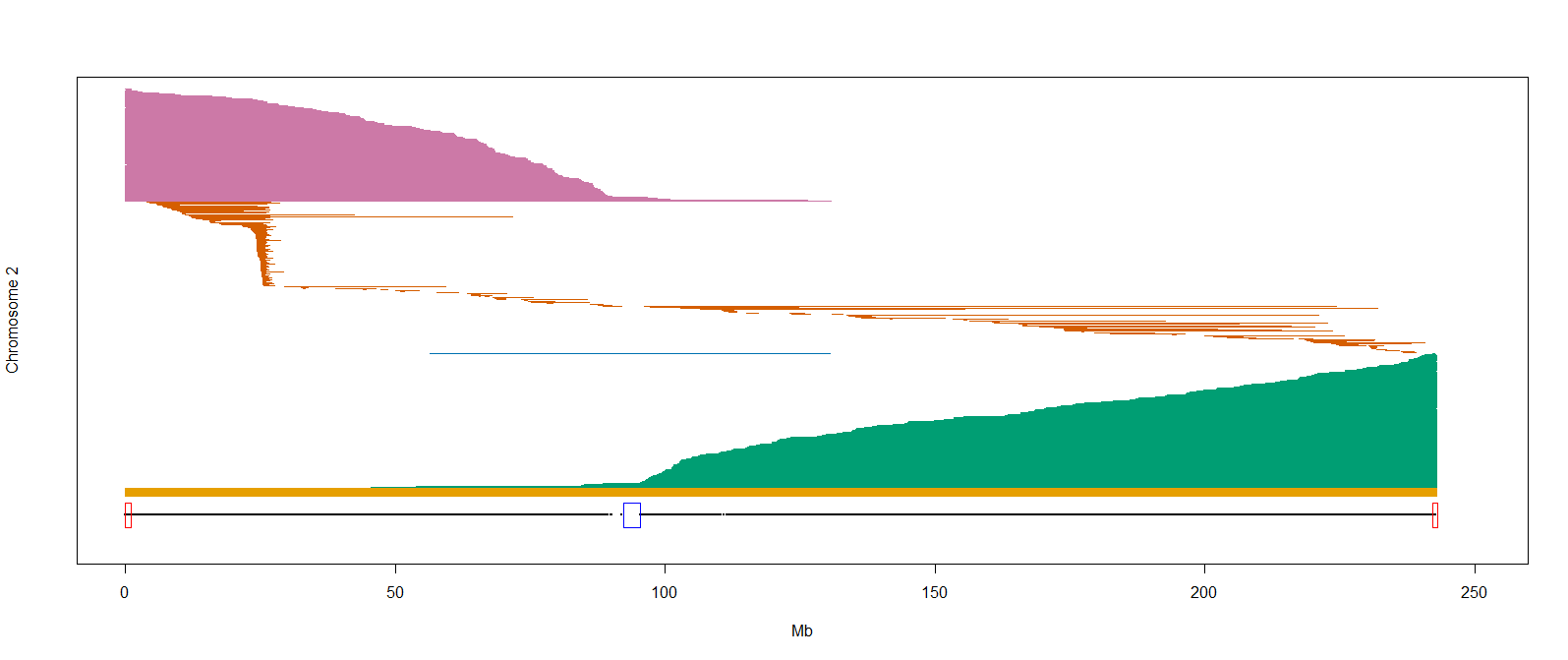


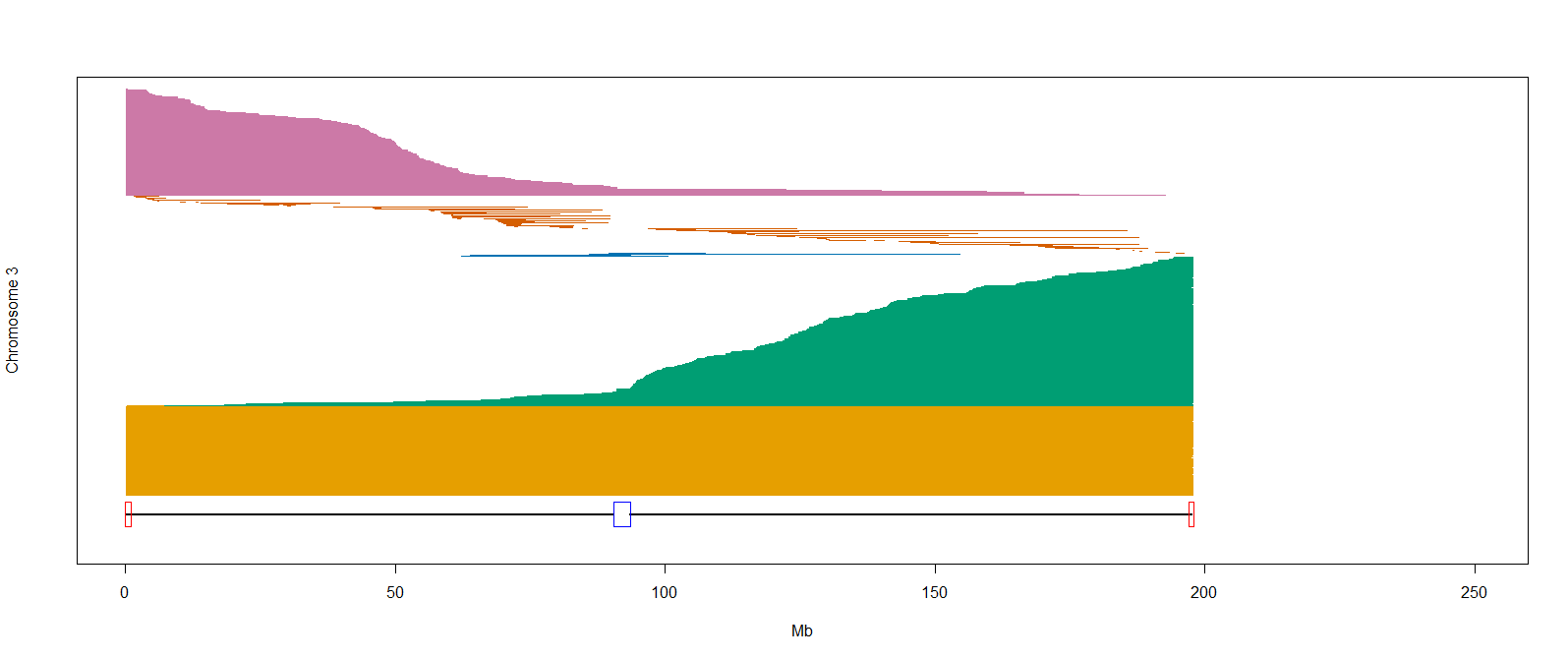


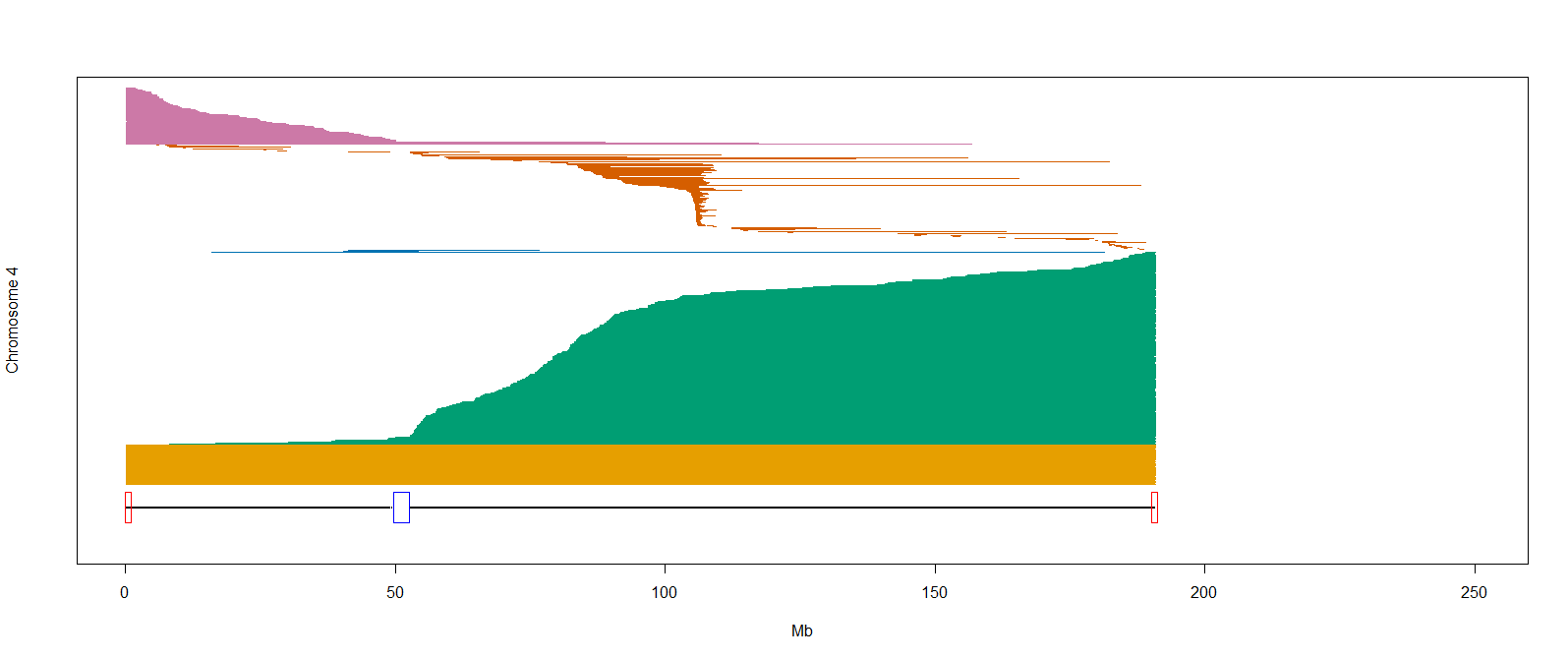


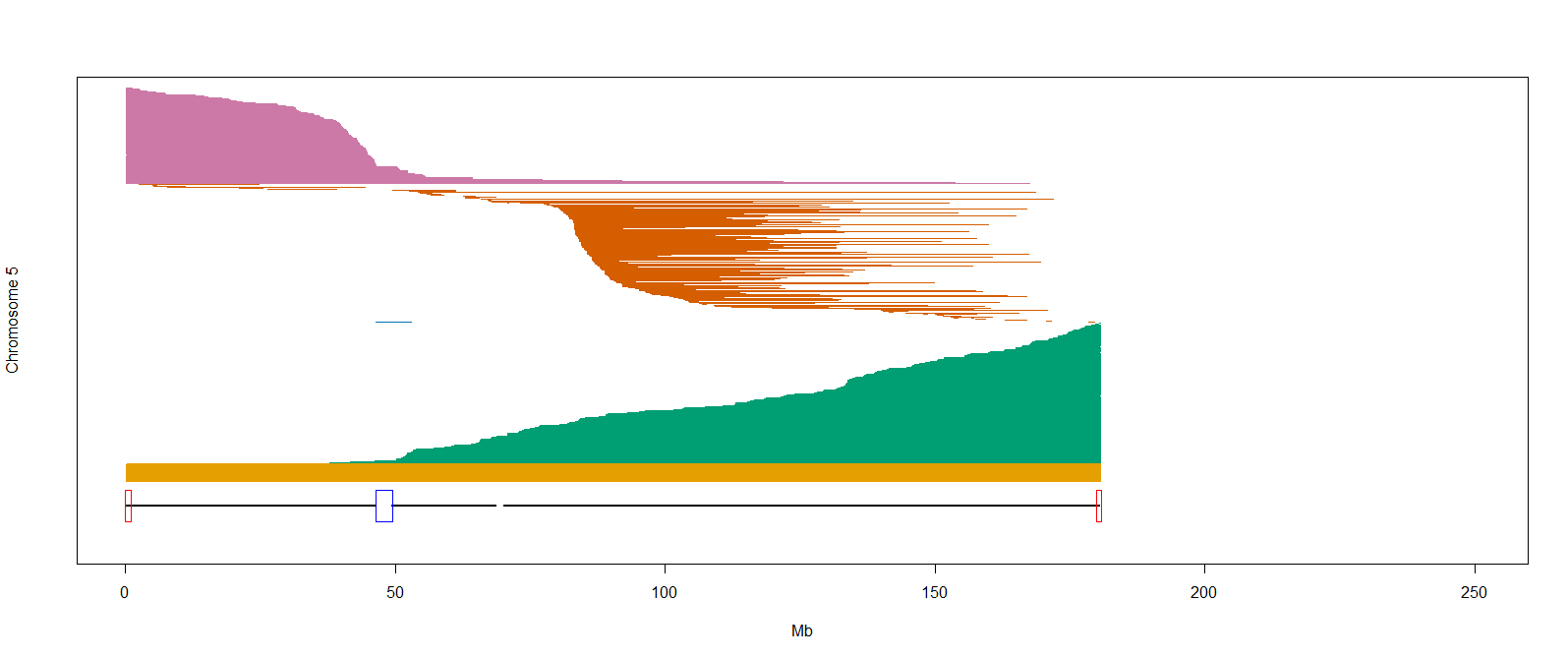


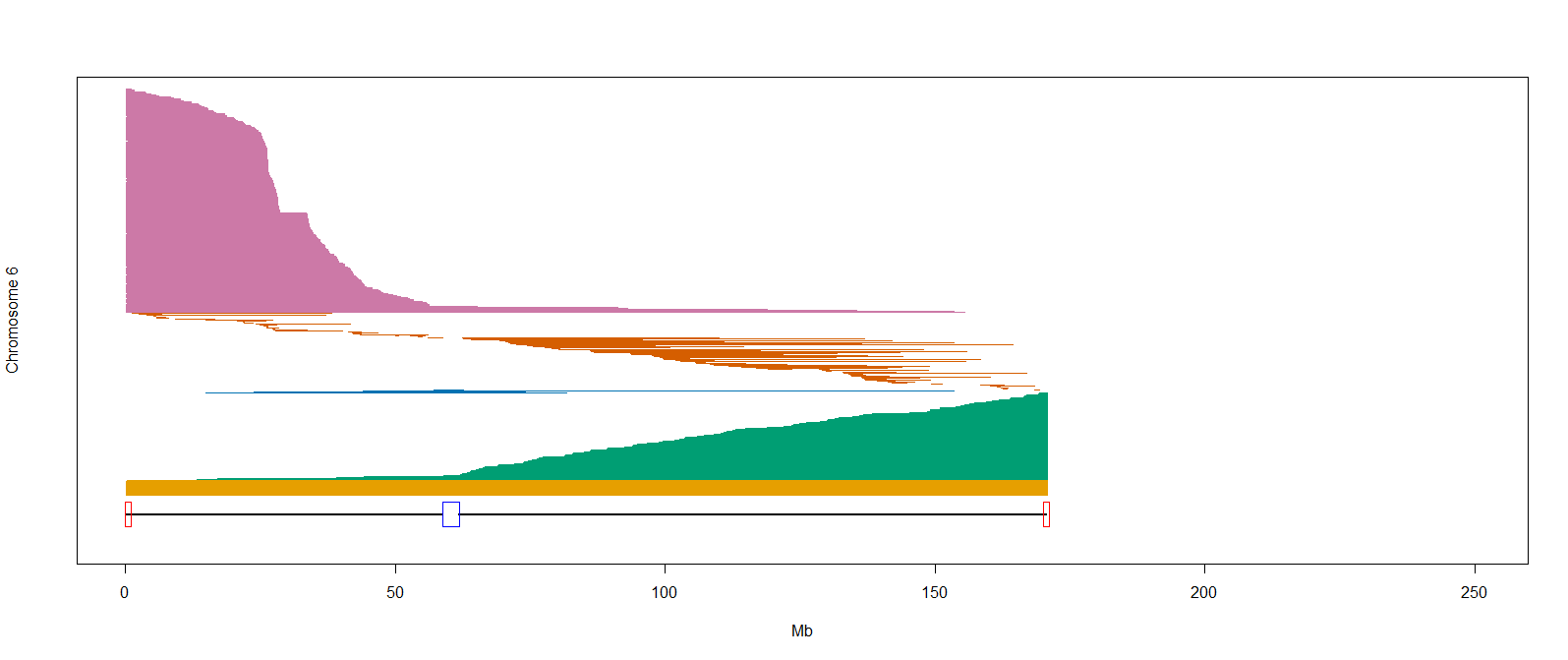


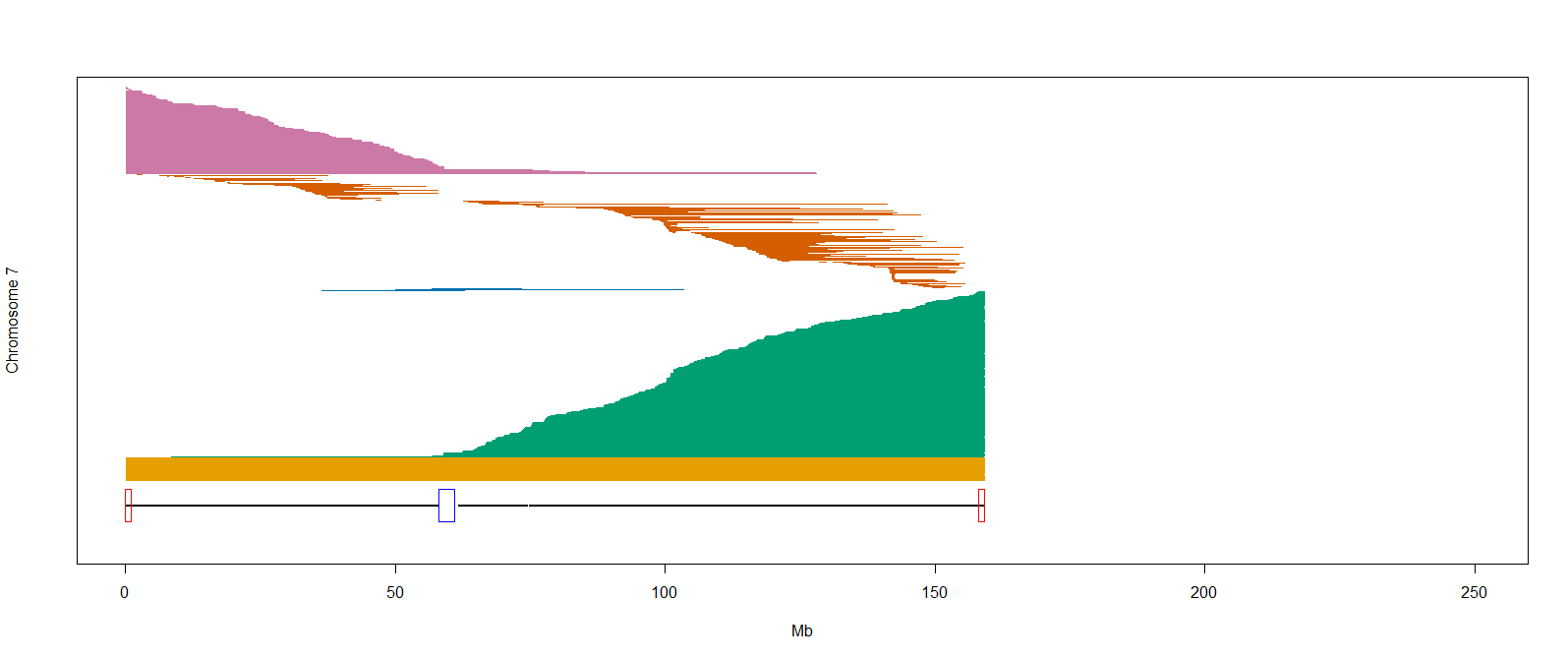


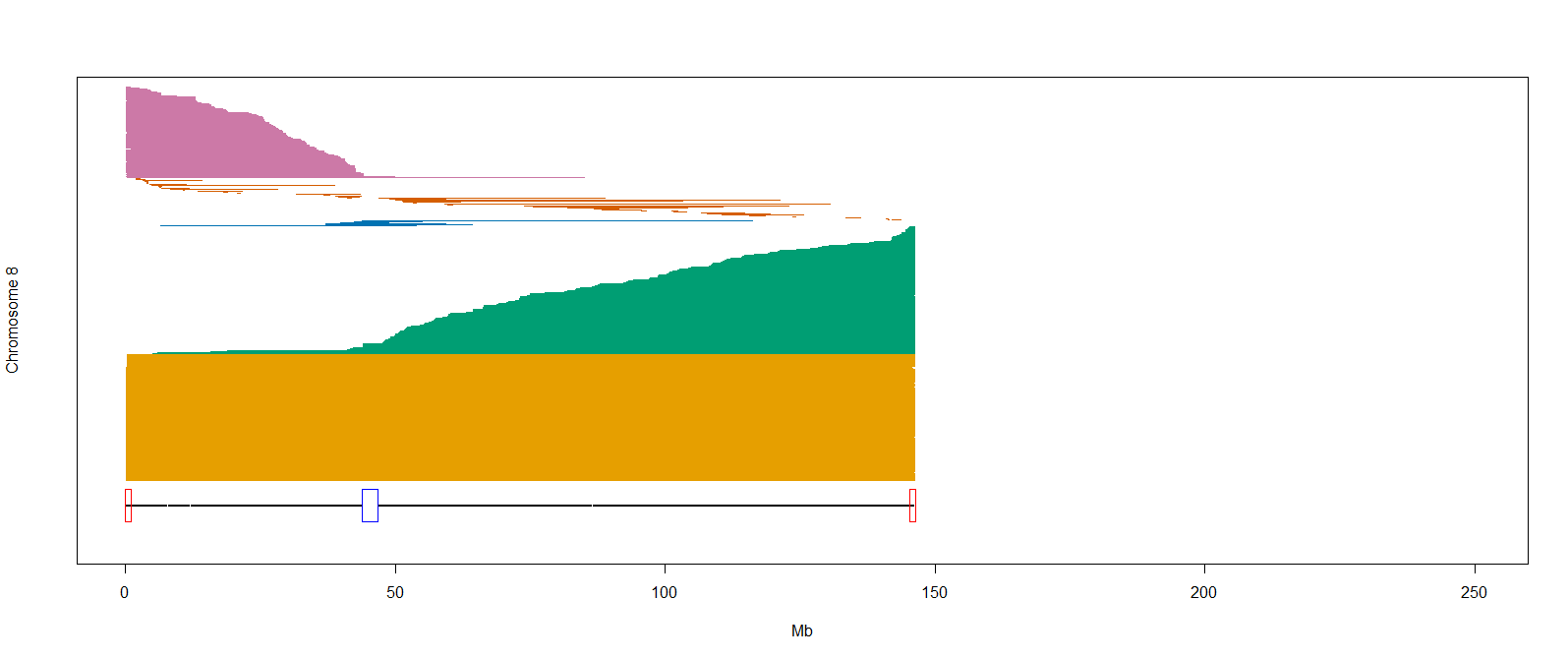


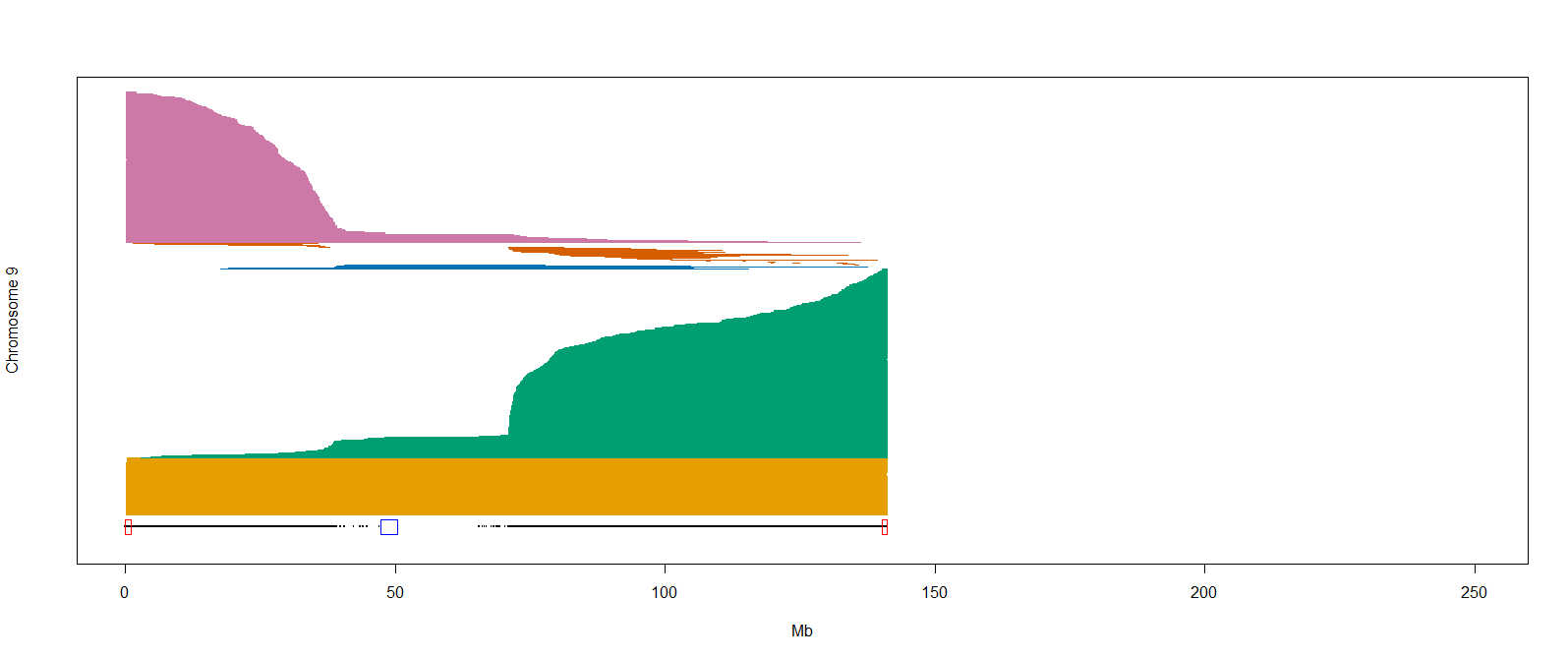


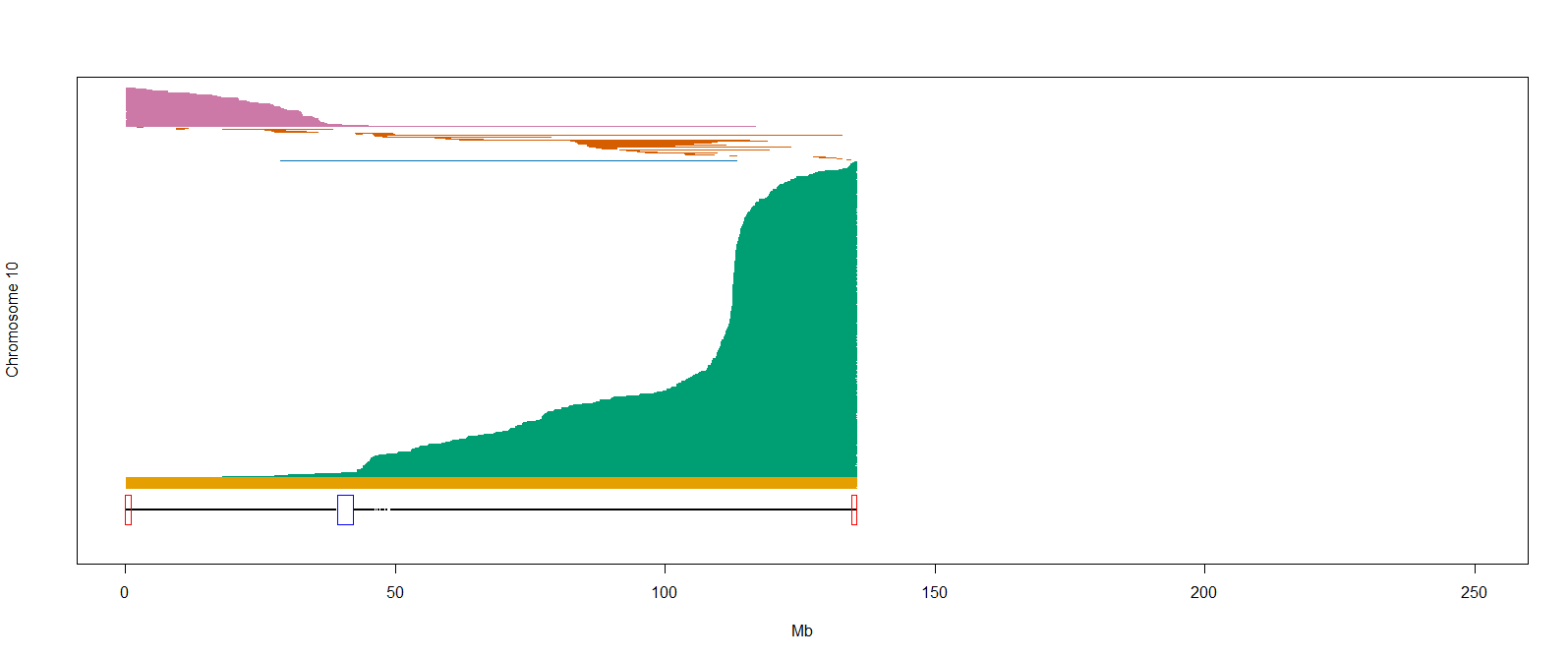


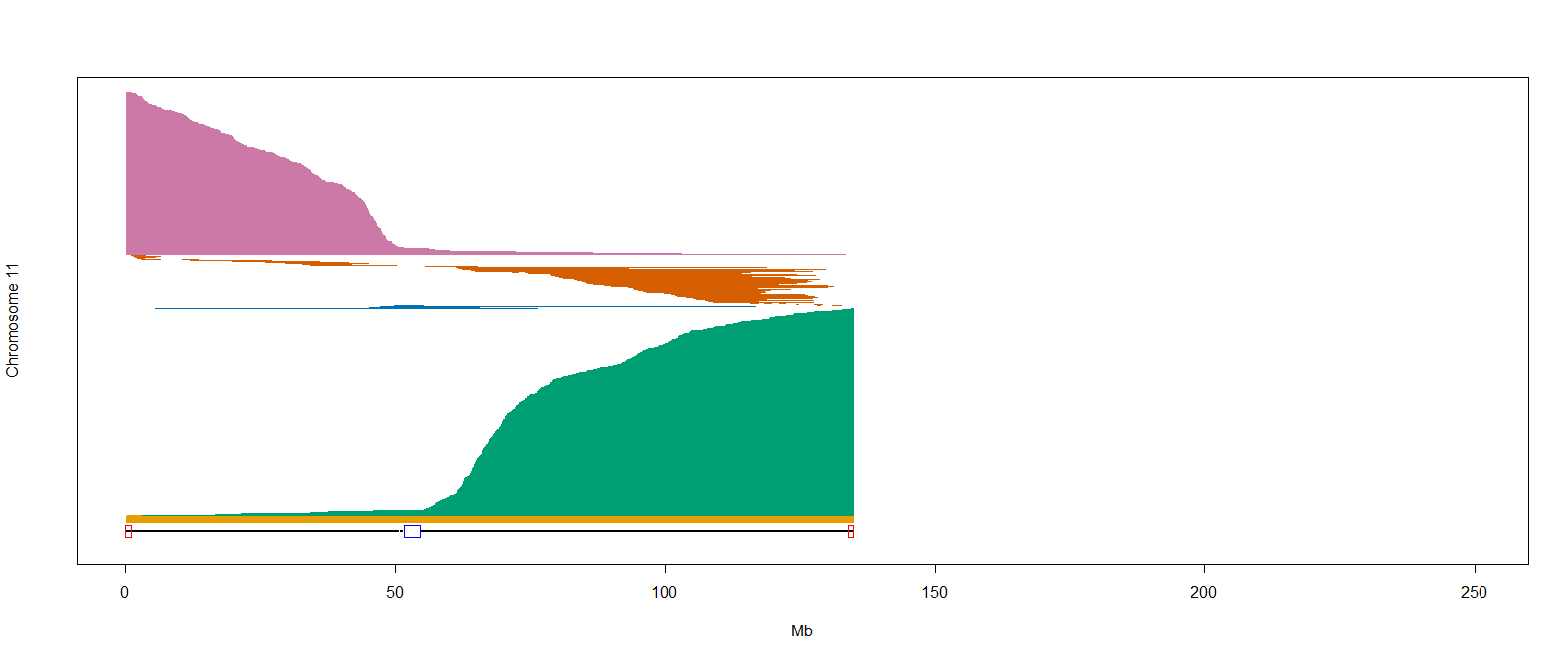


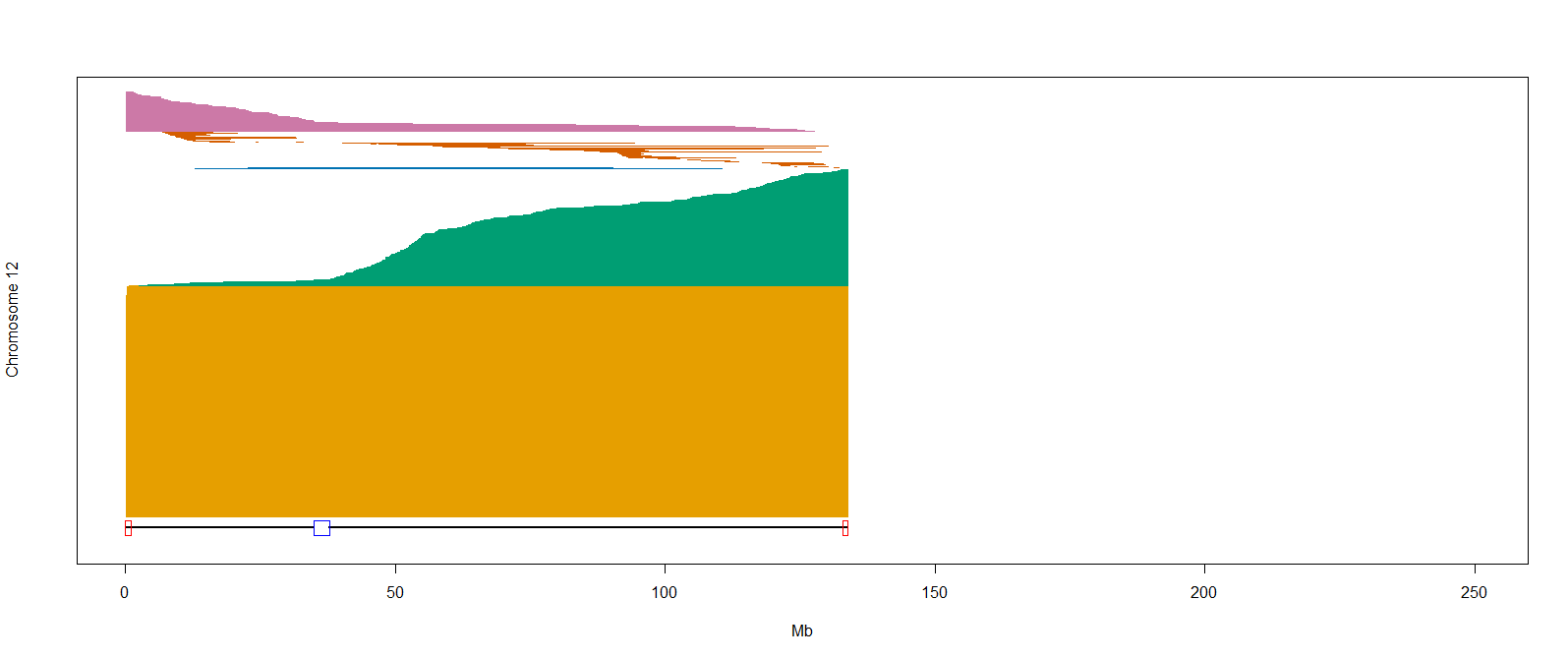


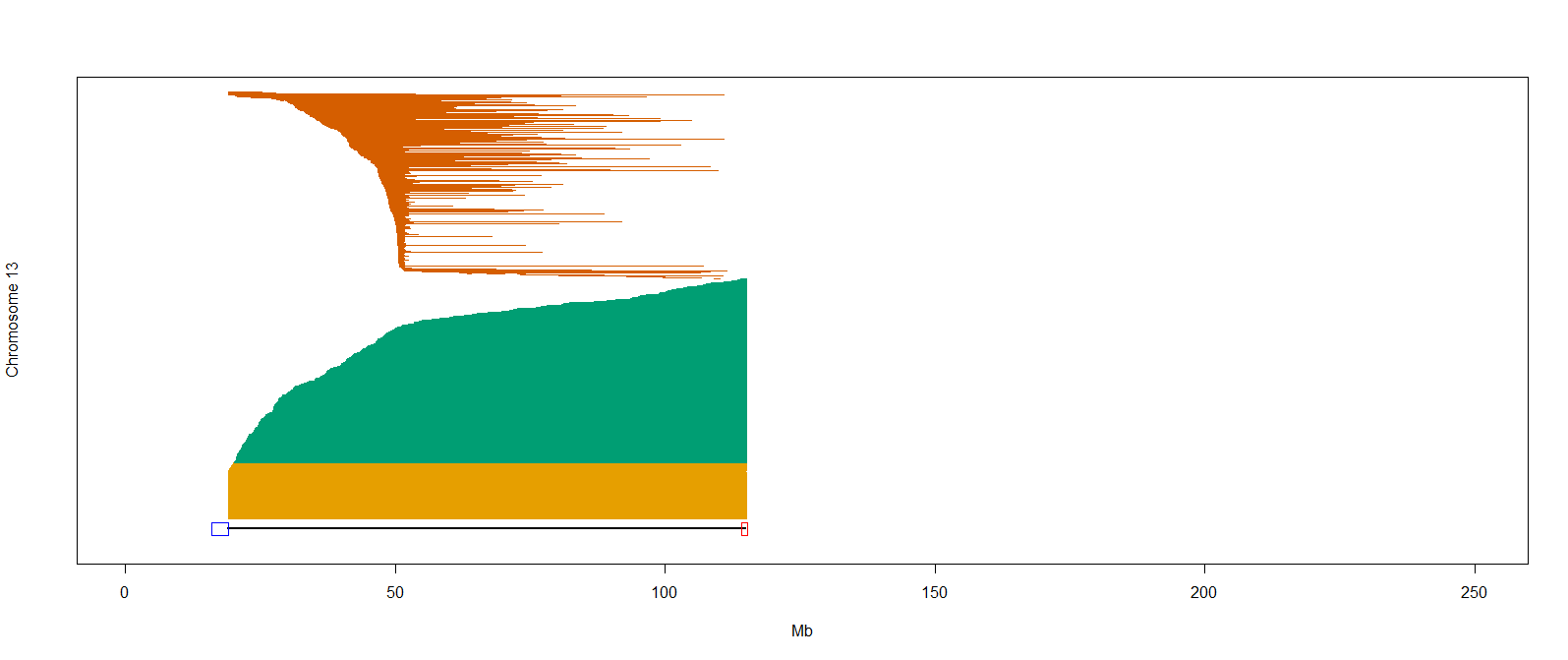


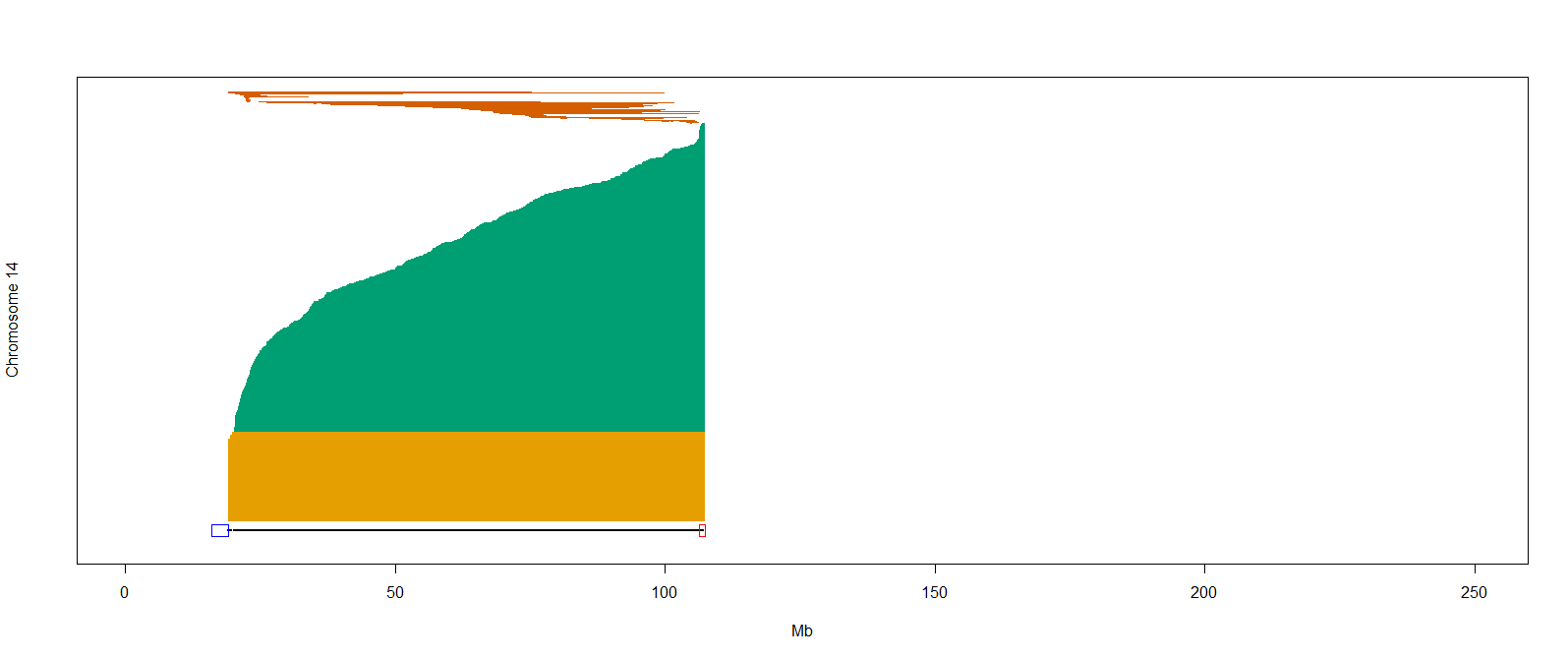


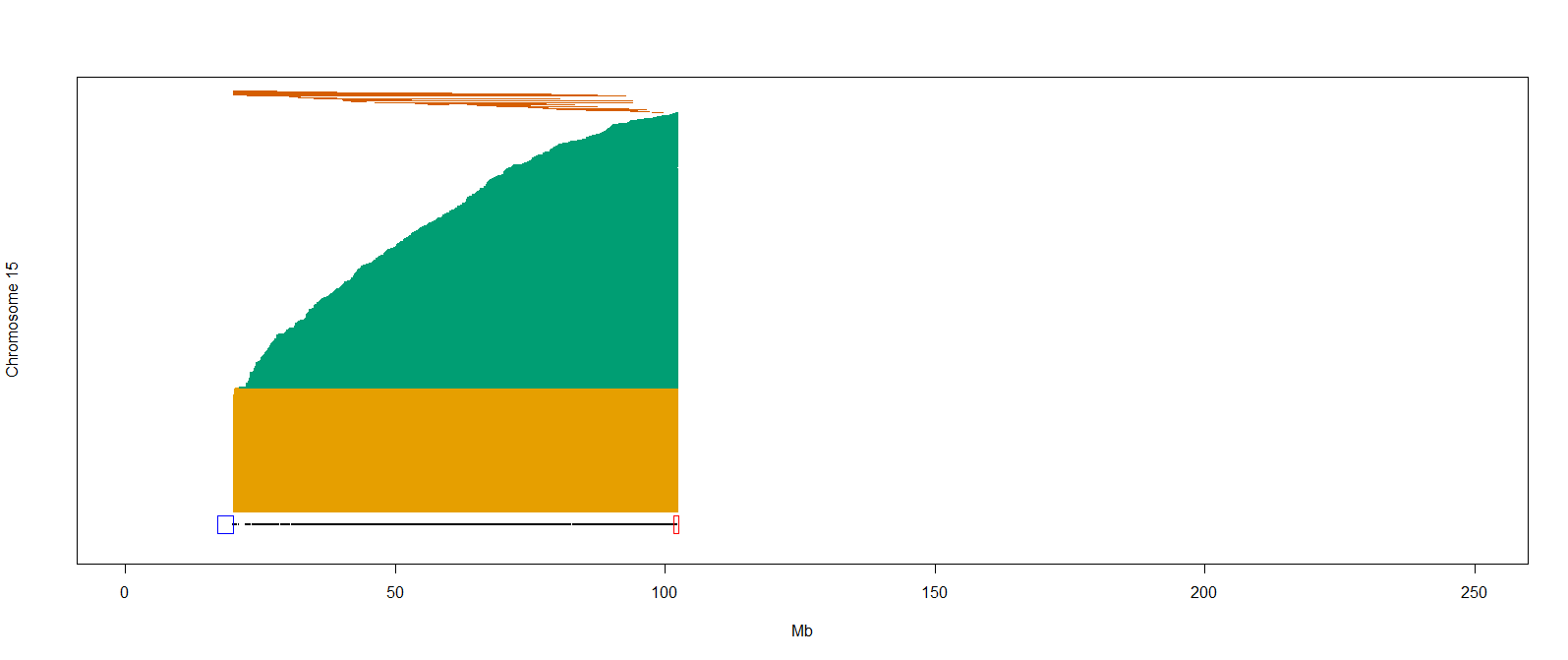


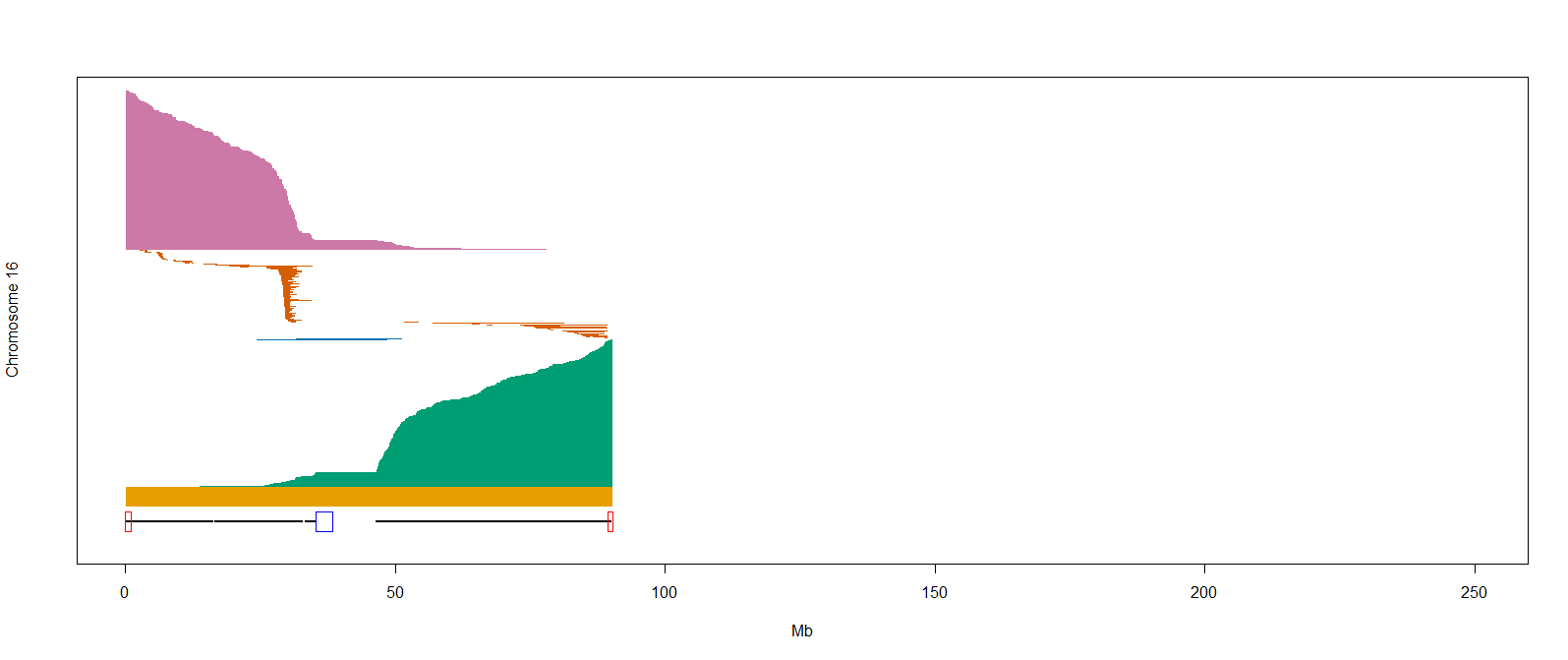


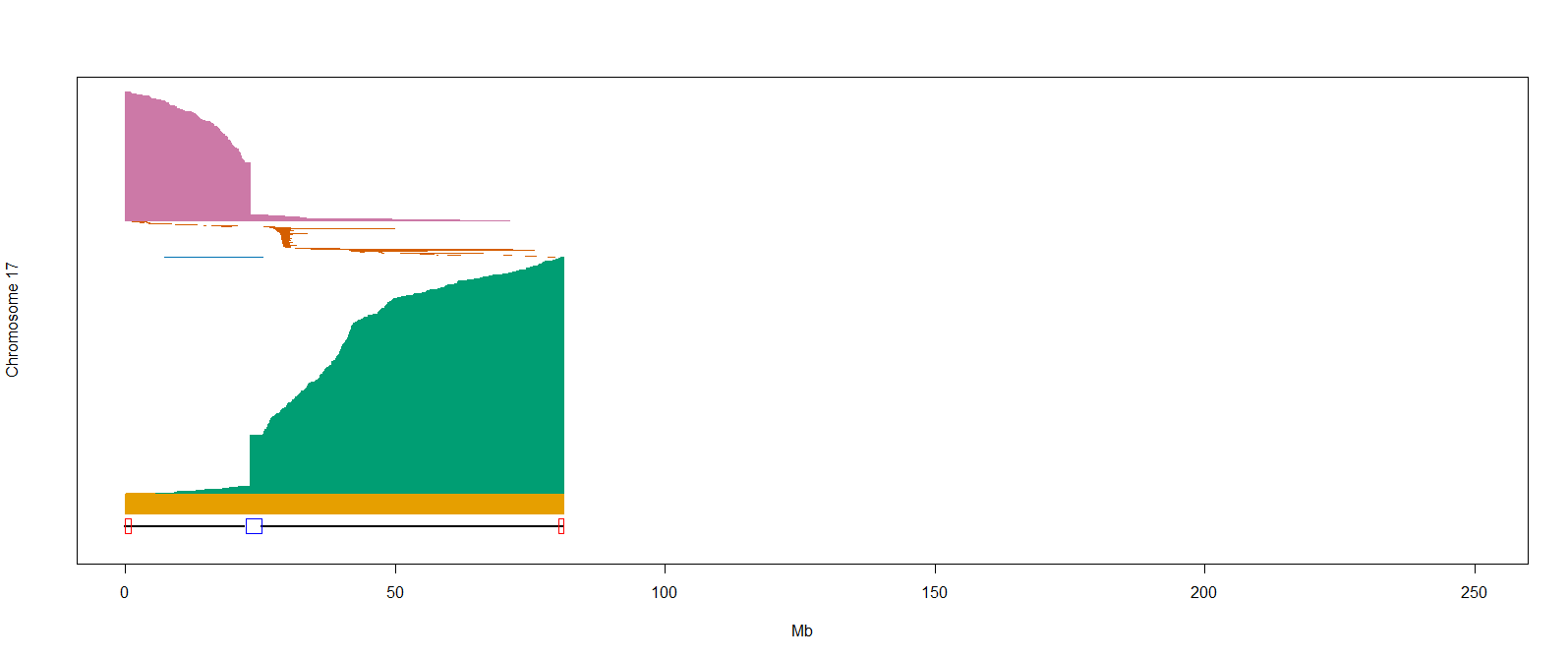


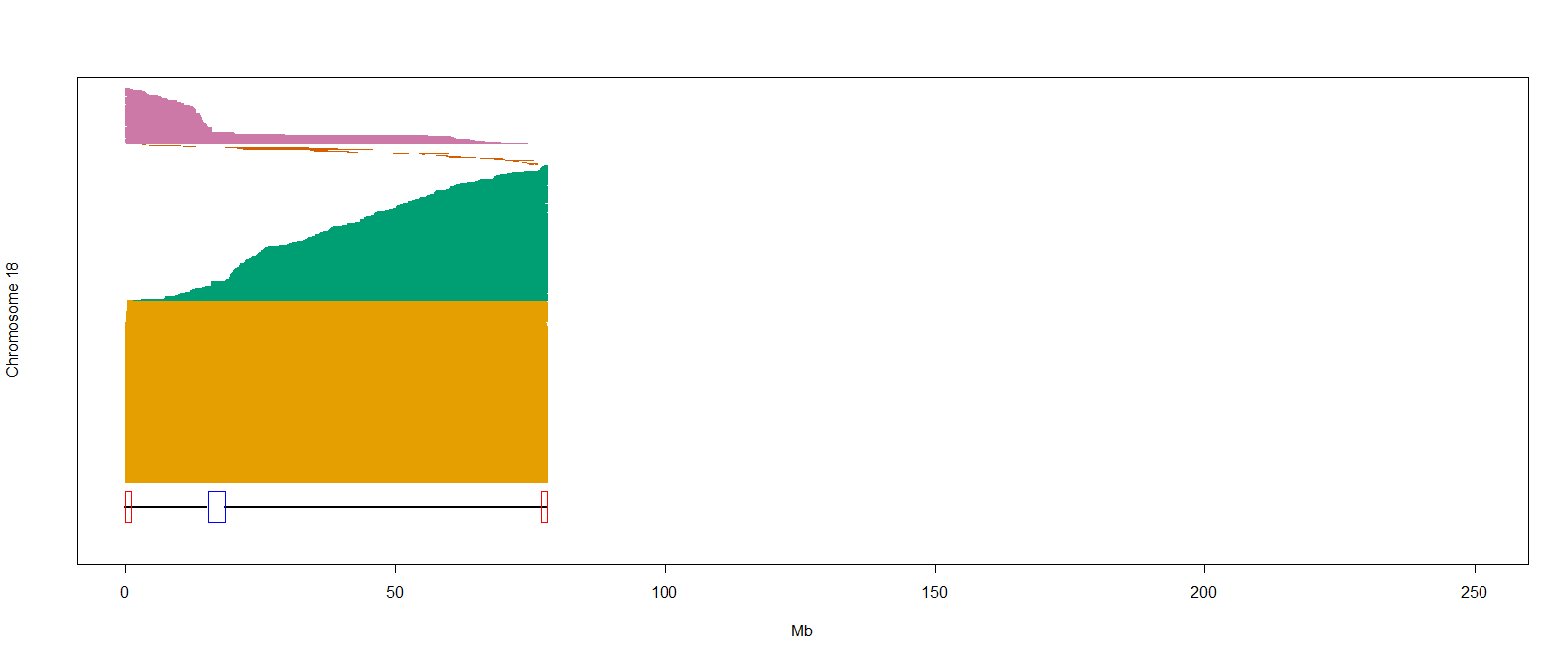


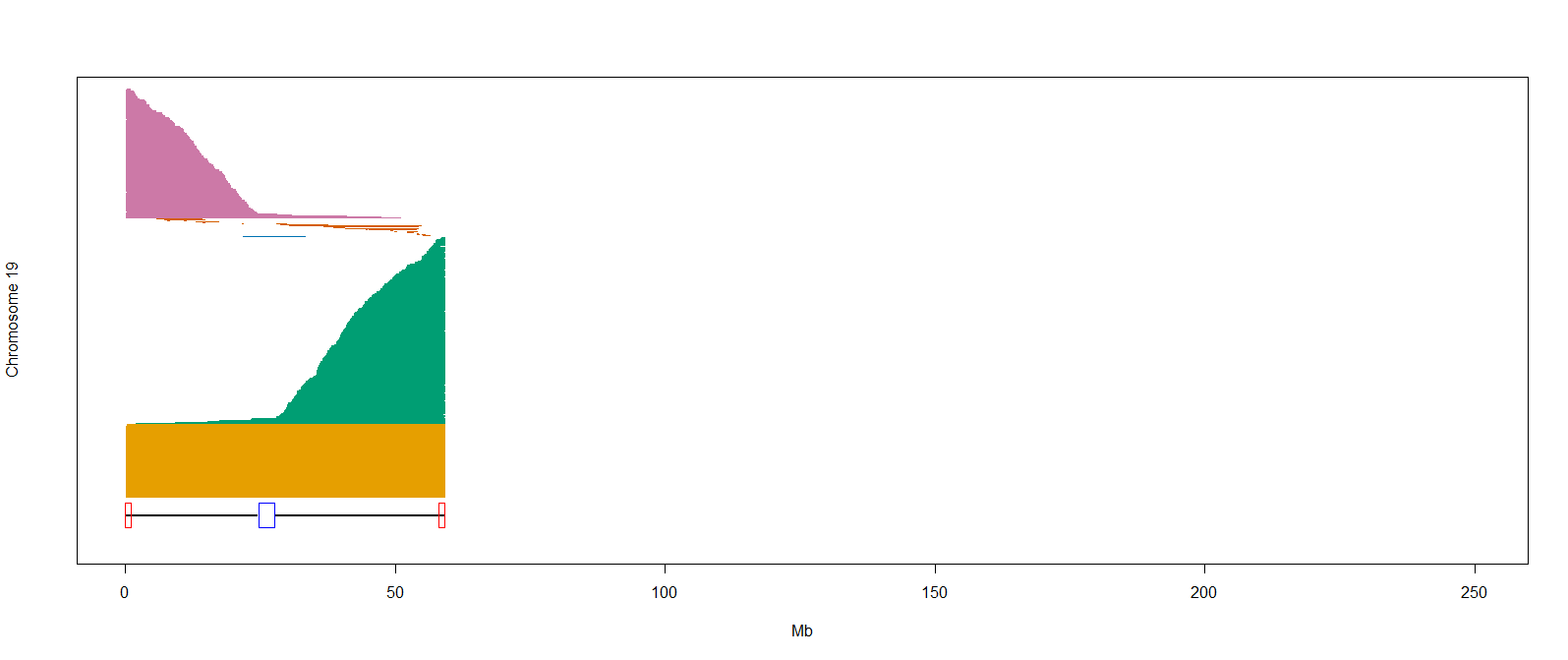


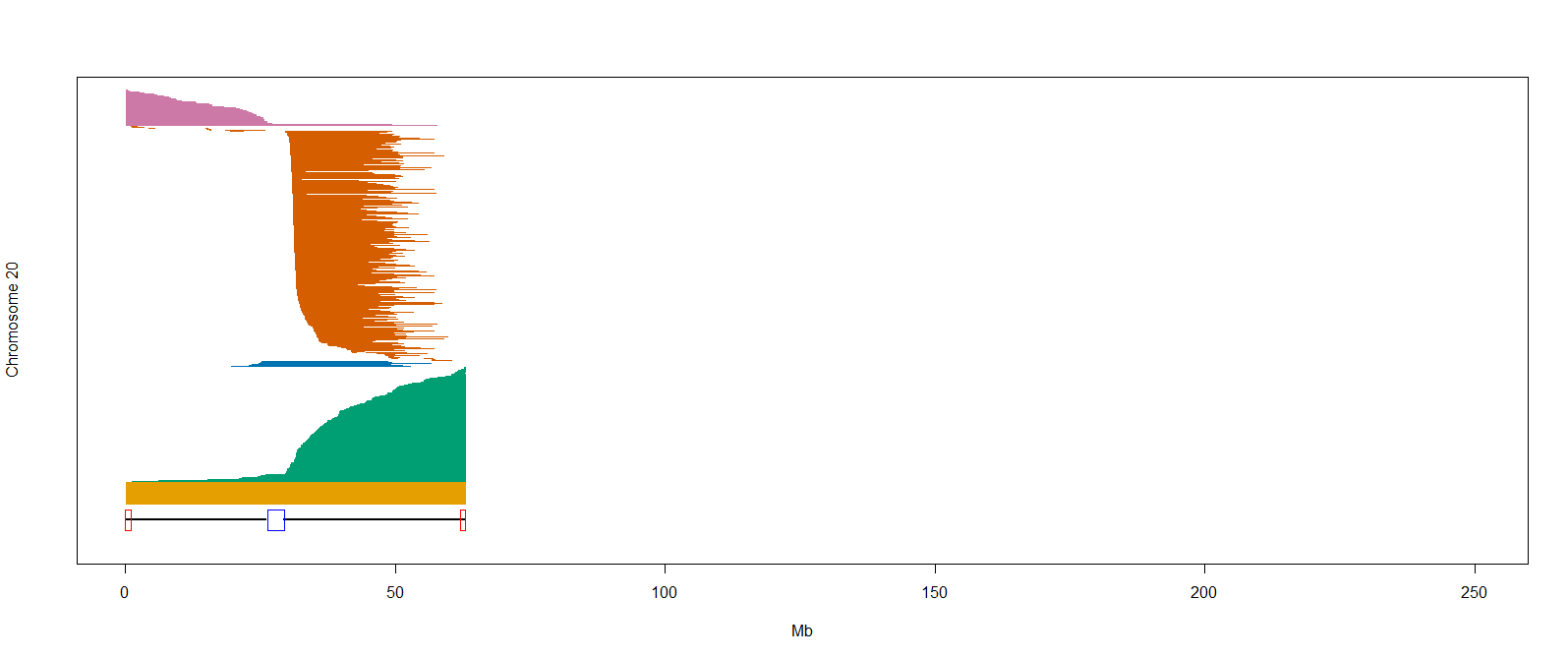


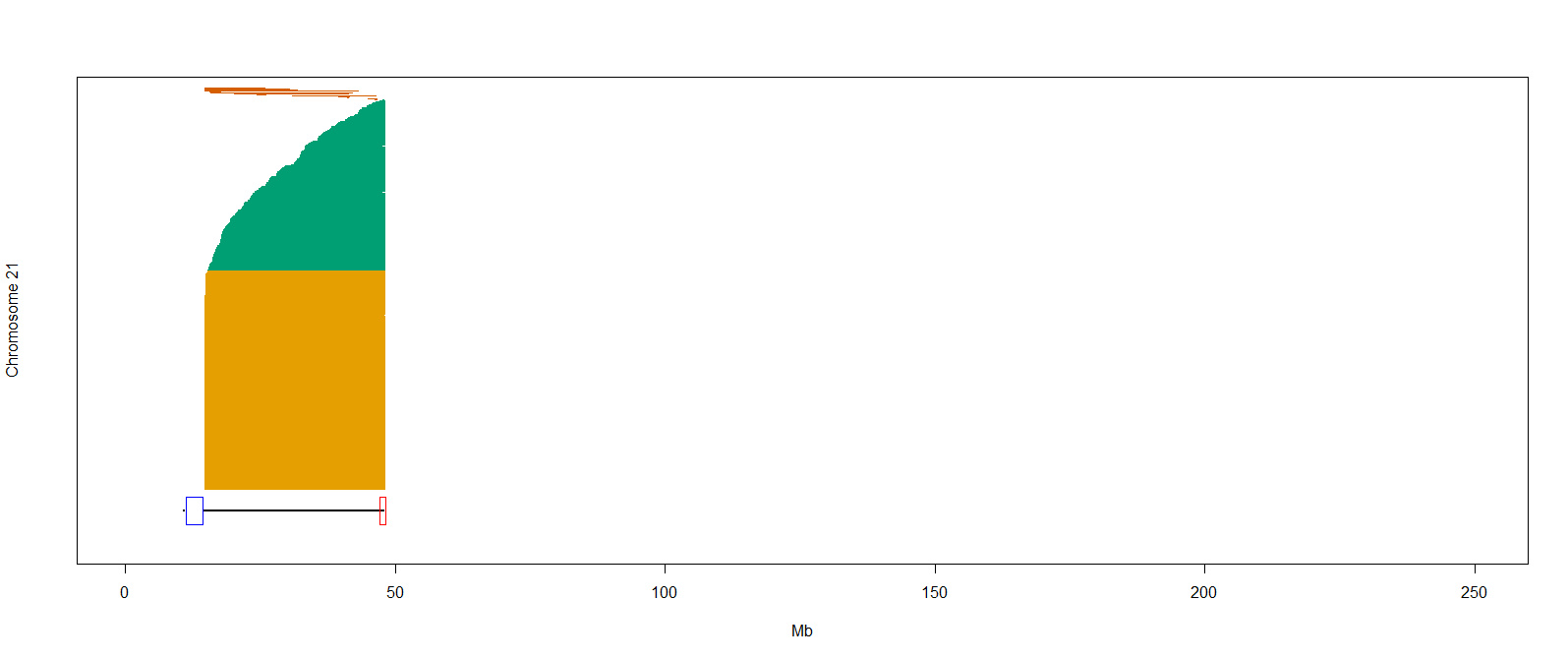


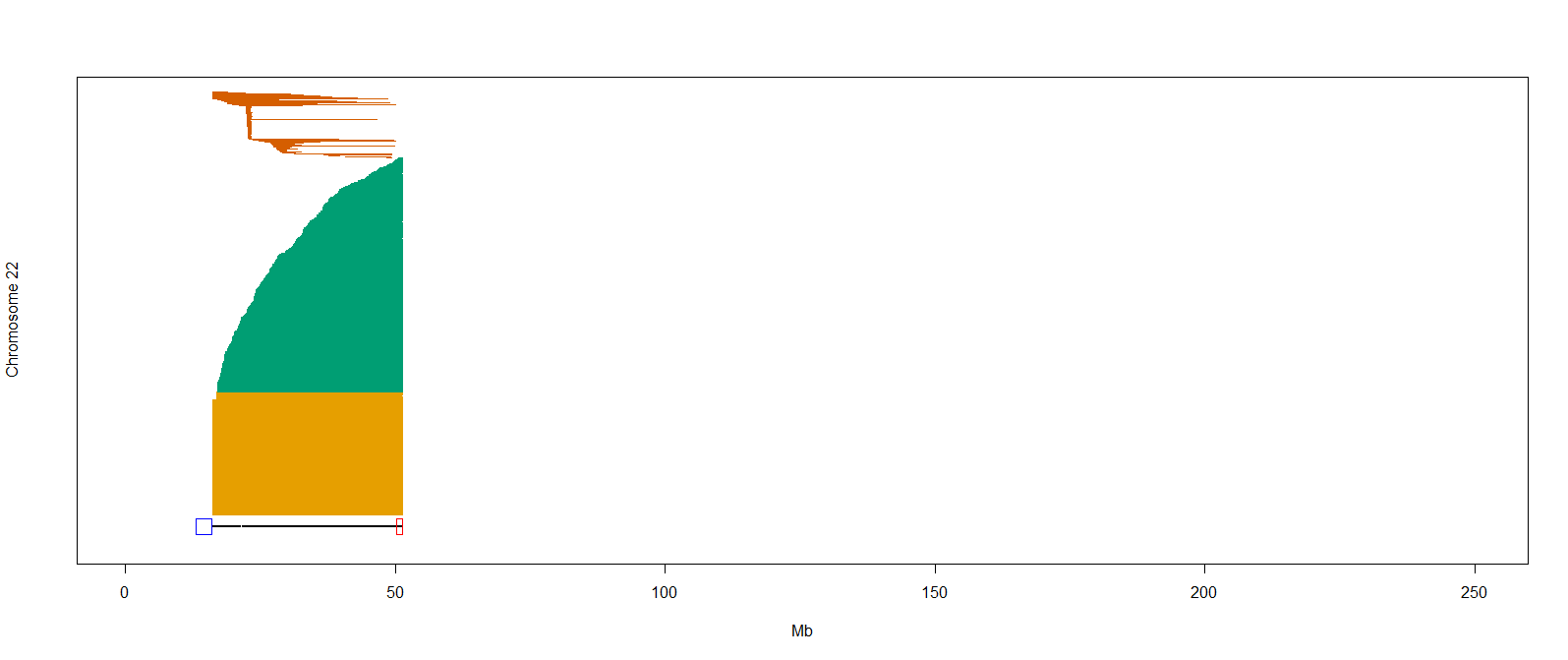


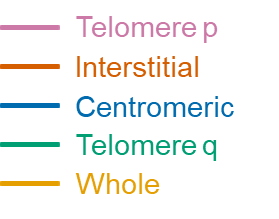

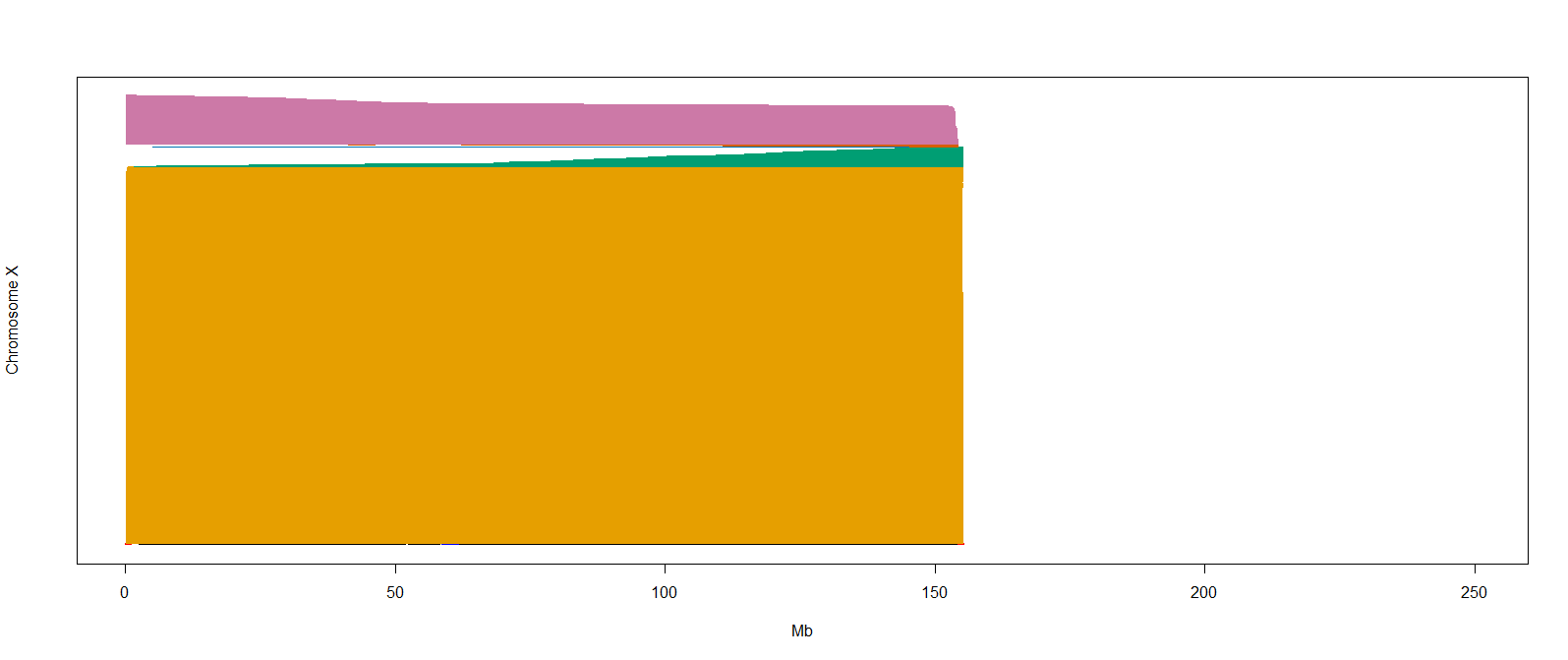


**
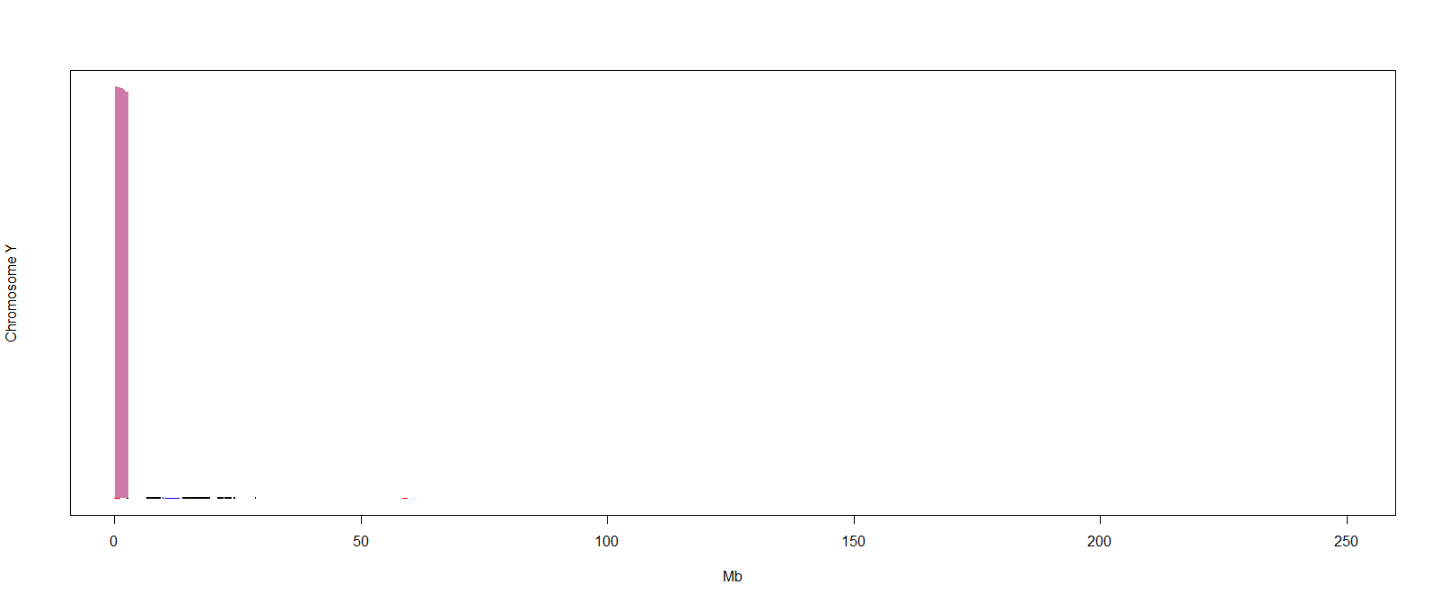
S2 Fig**. Categorization of clonal SCNAs by chromosome event location (e.g., telomeric ends, centromeric, interstitial, or whole chromosome event).
