# Supplementary material for: Genetically predicted telomere length is associated with clonal somatic copy number alterations in peripheral leukocytes
Source: PLoS Genet. 2020 Oct 22;16(10):e1009078. doi: 10.1371/journal.pgen.1009078 (PMC7608979; doi:10.1371/journal.pgen.1009078)
Supplement: S3 Fig — Plot (a) contains all 20 telomere length-associated variants from Li et al. (2020); plot (b) removes pleiotropic variants as detailed in S5 Table. (DOCX) [file pgen.1009078.s003.docx]

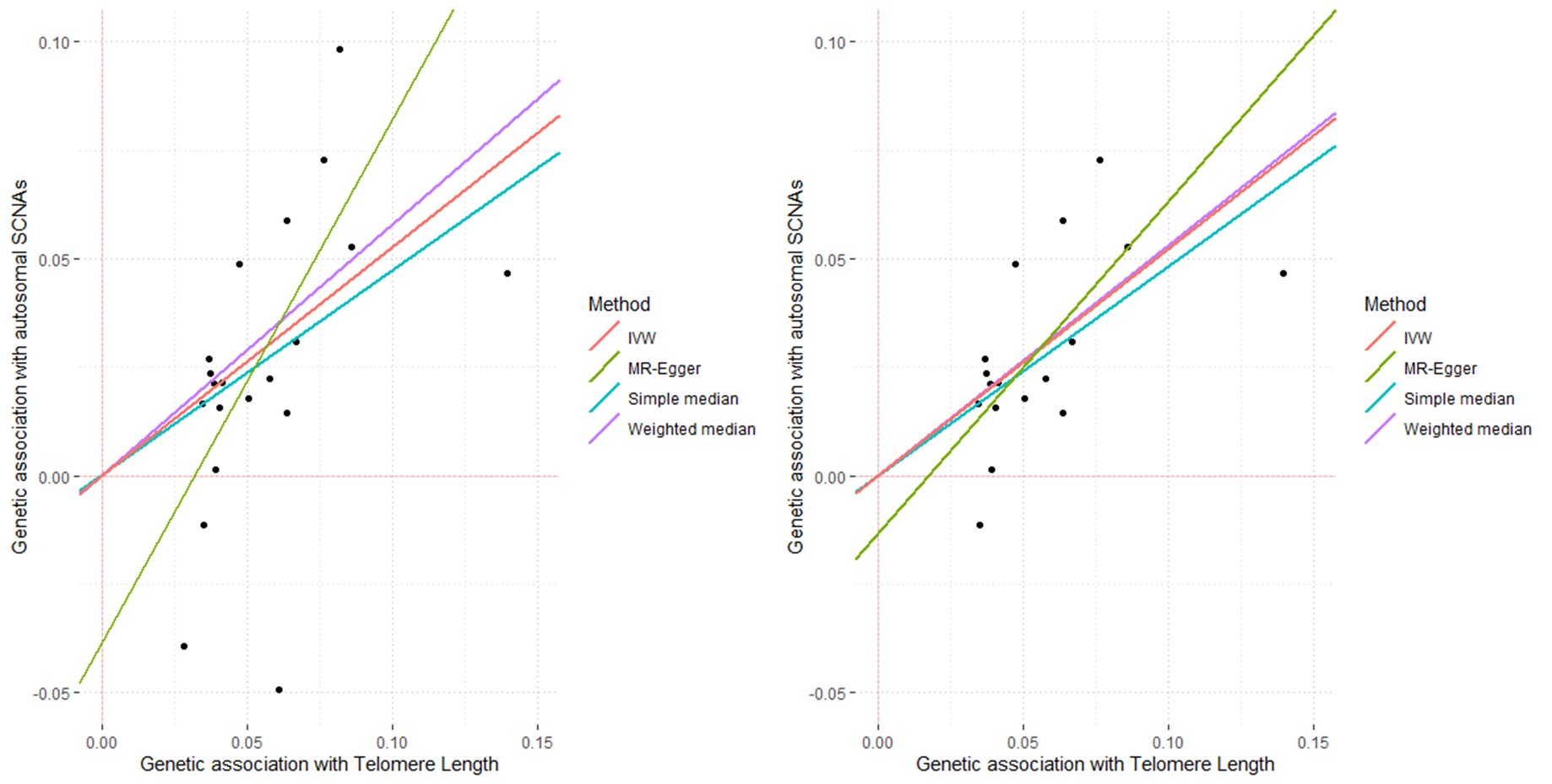


a.

b.

**S3 Fig**. Mendelian randomization estimates between telomere length associated variants and autosomal somatic copy number alterations. Plot (a) contains all 20 telomere length-associated variants from Li et al. (2020); plot (b) removes pleiotropic variants as detailed in **S5 Table**.
