# Supplementary material for: Genetically predicted telomere length is associated with clonal somatic copy number alterations in peripheral leukocytes
Source: PLoS Genet. 2020 Oct 22;16(10):e1009078. doi: 10.1371/journal.pgen.1009078 (PMC7608979; doi:10.1371/journal.pgen.1009078)
Supplement: S1 Table — (DOCX) [file pgen.1009078.s004.docx]

| **S1 Table**. UK Biobank population proportion breakdown by age and ethnicity | | | | | |
| --- | --- | --- | --- | --- | --- |
|  | Ethnicity | | | | |
| Age Quartile, % | White | Black | Asian | Other | Missing |
| ≤50 | 90.2 | 3.0 | 3.6 | 2.7 | 0.5 |
| 51-58 | 94 | 1.6 | 2.4 | 1.6 | 0.4 |
| 59-63 | 96.6 | 0.7 | 1.4 | 0.9 | 0.4 |
| ≥64 | 96.6 | 0.8 | 1.4 | 0.7 | 0.4 |
