# Supplementary material for: Genetically predicted telomere length is associated with clonal somatic copy number alterations in peripheral leukocytes
Source: PLoS Genet. 2020 Oct 22;16(10):e1009078. doi: 10.1371/journal.pgen.1009078 (PMC7608979; doi:10.1371/journal.pgen.1009078)
Supplement: S2 Table — (DOCX) [file pgen.1009078.s005.docx]

| **S2 Table**. Genetically-predicted telomere length by age and ethnicity | | | | | |
| --- | --- | --- | --- | --- | --- |
|  | Ethnicity | | | | |
| Age Quartile, Mean (SD) | White | Black | Asian | Other | Missing |
| ≤50 | -0.03 (0.99) | 1.07 (0.86) | 0.36 (1.01) | 0.38 (1.02) | 0.29 (1.08) |
| 51-58 | -0.03 (0.99) | 1.06 (0.89) | 0.38 (0.99) | 0.35 (1.06) | 0.15 (1.01) |
| 59-63 | -0.03 (0.99) | 1.11 (0.87) | 0.40 (1.01) | 0.30 (1.09) | 0.02 (1.08) |
| ≥64 | -0.04 (0.98) | 1.02 (0.89) | 0.36 (0.99) | 0.40 (1.04) | 0.10 (1.01) |
