# Supplementary material for: Genetically predicted telomere length is associated with clonal somatic copy number alterations in peripheral leukocytes
Source: PLoS Genet. 2020 Oct 22;16(10):e1009078. doi: 10.1371/journal.pgen.1009078 (PMC7608979; doi:10.1371/journal.pgen.1009078)
Supplement: S3 Table — (DOCX) [file pgen.1009078.s006.docx]

| **S3 Table**. Stratified associations between genetically-predicted telomere length and autosomal SCNAs | | | | | | |
| --- | --- | --- | --- | --- | --- | --- |
|  | Univariable Model | |  | Multivariable Model | |  |
|  | OR (95% CI) | p-value |  | OR (95% CI) | p-value | p-value_het_^a^ |
| Sex |  |  |  |  |  | 0.8588 |
| Male | 1.05 (1.029-1.077) | 1.06x10^-5^ |  | 1.07 (1.046-1.096) | 9.18x10^-9^ |  |
| Female | 1.05 (1.023-1.071) | 7.73x10^-5^ |  | 1.07 (1.043-1.093) | 3.62x10^-8^ |  |
| Age Quartile |  |  |  |  |  | 0.2207 |
| ≤50 | 1.01 (0.970-1.054) | 0.5886 |  | 1.03 (0.984-1.071) | 0.2219 |  |
| 51-58 | 1.05 (1.018-1.091) | 0.0029 |  | 1.07 (1.030-1.105) | 2.95x10^-4^ |  |
| 59-63 | 1.07 (1.039-1.105) | 1.23x10^-5^ |  | 1.08 (1.047-1.114) | 1.33x10^-6^ |  |
| ≥64 | 1.07 (1.042-1.101) | 1.15x10^-6^ |  | 1.08 (1.051-1.110) | 5.26x10^-8^ |  |
| Smoking Status |  |  |  |  |  | 0.4056 |
| Never | 1.04 (1.018-1.065) | 5.24x10^-4^ |  | 1.06 (1.039-1.088) | 2.33x10^-7^ |  |
| Former | 1.07 (1.047-1.104) | 1.04x10^-7^ |  | 1.09 (1.058-1.116) | 1.50x10^-9^ |  |
| Current | 1.03 (0.978-1.078) | 0.2903 |  | 1.04 (0.994-1.097) | 0.0840 |  |
| Missing | 0.98 (0.836-1.139) | 0.7550 |  | 1.01 (0.862-1.189) | 0.8792 |  |
| Ethnicity |  |  |  |  |  | 0.1484 |
| White | 1.07 (1.054-1.089) | 6.34x10^-16^ |  | 1.07 (1.055-1.091) | <2x10^-16^ |  |
| Black | 1.03 (0.845-1.225) | 0.7732 |  | 1.03 (0.845-1.258) | 0.7620 |  |
| Asian | 0.96 (0.839-1.107) | 0.6008 |  | 0.96 (0.835-1.103) | 0.5633 |  |
| Other | 0.91 (0.779-1.059) | 0.2194 |  | 0.92 (0.788-1.072) | 0.2851 |  |
| Missing | 0.92 (0.710-1.183) | 0.5009 |  | 0.98 (0.748-273) | 0.8586 |  |
| Multivariable models control for sex, age, age^2^, genetic ancestry, and detailed smoking status | | | | | |  |
| ^a^Denotes test of heterogeneity | |  |  |  |  |  |
| age^2^= age-squared | |  |  |  |  |  |
