# Supplementary material for: Genetically predicted telomere length is associated with clonal somatic copy number alterations in peripheral leukocytes
Source: PLoS Genet. 2020 Oct 22;16(10):e1009078. doi: 10.1371/journal.pgen.1009078 (PMC7608979; doi:10.1371/journal.pgen.1009078)
Supplement: S4 Table — (DOCX) [file pgen.1009078.s007.docx]

| **S4 Table**. Associations of telomere length-associated variants from Taub et al. (2019) with autosomal SCNAs | | | | | | | | | | | | |
| --- | --- | --- | --- | --- | --- | --- | --- | --- | --- | --- | --- | --- |
|  |  |  |  |  |  |  |  |  | **Telomere Length Association**^a^ |  | **Association with Autosomal SCNAs**^b^ | |
| **Nearby gene** | **CHR** | **Position (hg37)** | **SNP** | **Ref** | **Alt** | **UKBB AAF** | **TOPMed AAF** |  | **BP** |  | **β** | **p-value** |
| ACYP2 | 2 | 54495222 | rs7579722 | G | C | 0.15 | 0.15 |  | 37.5 |  | 0.00046 | 0.9775 |
| RPN1 | 3 | 128422176 | rs60092972 | A | T | 0.31 | 0.32 |  | 23.6 |  | 0.00541 | 0.6720 |
| TERC | 3 | 169482335 | rs2293607 | C | T | 0.76 | 0.76 |  | 70.7 |  | 0.05814 | 2.72X10^-5^ |
| NAF1 | 4 | 164048199 | rs4691895 | G | C | 0.78 | 0.76 |  | 39.5 |  | 0.02524 | 0.0736 |
| TERT | 5 | 1285974 | rs7705526 | C | A | 0.32 | 0.33 |  | 60 |  | 0.09781 | 1.11X10^-14^ |
| POT1 | 7 | 124494861 | rs10246424 | G | A | 0.73 | 0.72 |  | 28.8 |  | 0.01712 | 0.1924 |
| OPRK1 | 8 | 54434760 | rs188891454 | C | T | 1.00 | 1.00 |  | 234 |  | -0.10545 | 0.3827 |
| LINC01592 | 8 | 70243701 | rs144510686 | T | G | 1.00 | 1.00 |  | 382.3 |  | 0.0231 | 0.9236 |
| TERF1 | 8 | 73950559 | rs12679652 | A | G | 0.32 | 0.32 |  | 28.8 |  | -0.00457 | 0.7161 |
| SH3PXD2A | 10 | 105679341 | rs2488002 | T | C | 0.17 | 0.17 |  | 64.3 |  | 0.04342 | 0.0059 |
| TINF2 | 14 | 24711798 | rs28372734 | C | G | 0.00 | 0.00 |  | 152.1 |  | 0.0222 | 0.9370 |
| DCAF4 | 14 | 73432100 | rs78517833 | A | T | 0.10 | 0.10 |  | 36.8 |  | 0.04748 | 0.0126 |
| TCL1A | 14 | 96180685 | rs11846938 | T | G | 0.24 | 0.24 |  | 25.6 |  | -0.0433 | 0.0021 |
| TERF2 | 16 | 69391714 | rs9925619 | C | G | 0.29 | 0.28 |  | 26.8 |  | -0.00637 | 0.6222 |
| RFWD3 | 16 | 74676964 | rs28616016 | C | T | 0.58 | 0.58 |  | 31.4 |  | 0.04974 | 2.89X10^-5^ |
| ZNF676 | 19 | 22424997 | rs281173 | G | A | 0.62 | 0.63 |  | 22.3 |  | 0.02079 | 0.0855 |
| SAMHD1 | 20 | 35578680 | rs4810362 | G | A | 0.85 | 0.85 |  | 34.7 |  | 0.00729 | 0.6583 |
| LINC01429 | 20 | 50453984 | rs6091385 | C | T | 0.11 | 0.12 |  | 31.9 |  | -0.00367 | 0.8431 |
| RTEL1 | 20 | 62336258 | rs6062497 | T | C | 0.33 | 0.31 |  | 42 |  | 0.0149 | 0.2292 |
| CHKB | 22 | 51034870 | rs131742 | G | A | 0.62 | 0.62 |  | 26.1 |  | 0.01668 | 0.1706 |
| Multivariable models included the variant of interest and controlled for sex, age, age^2^, genetic ancestry, and detailed smoking status | | | | | | | | | | | | |
| ^a^Positive change in number of base pairs from Taub et al. (2019) | | | | | | | |  |  |  |  |  |
| ^b^β estimate for each variant from UK Biobank | | | | | | | | | | | | |
| AAF= Alternate allele frequency | | |  |  |  |  |  |  |  |  |  |  |
| BP= Base pairs | |  |  |  |  |  |  |  |  |  |  |  |
| age^2^= age-squared | |  |  |  |  |  |  |  |  |  |  |  |
