# Supplementary material for: Genetically predicted telomere length is associated with clonal somatic copy number alterations in peripheral leukocytes
Source: PLoS Genet. 2020 Oct 22;16(10):e1009078. doi: 10.1371/journal.pgen.1009078 (PMC7608979; doi:10.1371/journal.pgen.1009078)
Supplement: S5 Table — (DOCX) [file pgen.1009078.s008.docx]

| **S5 Table**. Associations of telomere length-associated variants from Li et al. (2020) with autosomal SCNAs | | | | | | | | | | | | | |
| --- | --- | --- | --- | --- | --- | --- | --- | --- | --- | --- | --- | --- | --- |
|  |  |  |  |  |  |  |  | **Telomere Length Association^a^** | |  | **Association with Autosomal SCNAs^b^** | |  |
| **Nearby gene** | **CHR** | **Position (hg37)** | **SNP** | **Ref** | **Alt** | **UKBB AAF** |  | β | SE |  | β | SE | FDR^c^ |
| PARP1 | 1 | 226562621 | rs3219104 | A | C | 0.85 |  | 0.0417 | 0.0064 |  | 0.0215 | 0.0163 | 0.9091 |
| TERC | 3 | 169514585 | rs10936600 | T | A | 0.76 |  | 0.0858 | 0.0057 |  | 0.0528 | 0.0137 | 0.8228 |
| NAF1 | 4 | 164048199 | rs4691895 | G | C | 0.78 |  | 0.0577 | 0.0061 |  | 0.0224 | 0.0141 | 0.8228 |
| TERT | 5 | 1285974 | rs7705526 | C | A | 0.32 |  | 0.0820 | 0.0058 |  | 0.0983 | 0.0126 | 0.0007 |
| TERT | 5 | 1287194 | rs2853677 | A | G | 0.42 |  | 0.0638 | 0.0055 |  | 0.0589 | 0.0117 | 0.8228 |
| POT1 | 7 | 124554267 | rs59294613 | A | C | 0.71 |  | 0.0407 | 0.0055 |  | 0.0157 | 0.0130 | 0.8228 |
| STN1 (OBFC1) | 10 | 105675946 | rs9419958 | C | T | 0.14 |  | 0.0636 | 0.0071 |  | 0.0145 | 0.0165 | 0.9091 |
| ATM | 11 | 108105593 | rs228595 | A | G | 0.59 |  | 0.0285 | 0.0050 |  | -0.0394 | 0.0118 | 0.0002 |
| DCAF4 | 14 | 73404752 | rs2302588 | G | C | 0.10 |  | 0.0476 | 0.0084 |  | 0.0487 | 0.0189 | 0.8228 |
| MPHOSPH6 | 16 | 82199980 | rs7194734 | T | C | 0.23 |  | 0.0369 | 0.0060 |  | 0.0268 | 0.0138 | 0.8228 |
| ZNF208 | 19 | 22215441 | rs8105767 | A | G | 0.29 |  | 0.0392 | 0.0054 |  | 0.0013 | 0.0128 | 0.8228 |
| RTEL1/STMN3 | 20 | 62269750 | rs75691080 | T | C | 0.92 |  | 0.0671 | 0.0089 |  | 0.0310 | 0.0214 | 0.9745 |
| RTEL1 | 20 | 62291599 | rs34978822 | G | C | 0.98 |  | 0.1397 | 0.0227 |  | 0.0467 | 0.0424 | 0.8228 |
| RTEL1/ZBTB46 | 20 | 62436398 | rs73624724 | T | C | 0.14 |  | 0.0507 | 0.0074 |  | 0.0178 | 0.0165 | 0.9745 |
| SENP7 | 3 | 101232093 | rs55749605 | A | C | 0.38 |  | 0.0373 | 0.0067 |  | 0.0235 | 0.0120 | 0.9091 |
| MOB1B | 4 | 71774347 | rs13137667 | T | C | 0.97 |  | 0.0765 | 0.0137 |  | 0.0728 | 0.0348 | 0.9895 |
| CARMIL1 | 6 | 25480328 | rs34991172 | G | T | 0.92 |  | 0.0608 | 0.0105 |  | -0.0493 | 0.0212 | 0.0007 |
| PRRC2A | 6 | 31587561 | rs2736176 | G | C | 0.28 |  | 0.0345 | 0.0055 |  | 0.0167 | 0.0129 | 0.8228 |
| TERF2 | 16 | 69406986 | rs3785074 | A | G | 0.29 |  | 0.0351 | 0.0056 |  | -0.0114 | 0.0129 | 0.2899 |
| RFWD3 | 16 | 74680074 | rs62053580 | G | A | 0.84 |  | 0.0389 | 0.0071 |  | 0.0211 | 0.0159 | 0.8228 |
| ^a^Summary statistics available from Li et al. (2020) | | | | | | | | | | | | | |
| ^b^Univariable estimates for each variant from UK Biobank | | | | | | | | | | | | | |
| AAF= Alternate allele frequency | | | | | | | | | | | | | |
| ^c^False discovery rate for each variant as calculated using GLIDE (Dai et al. (2018)). Three variants (rs7705526, rs228595, rs34991172) were detected to have evidence of pleiotropy (FDR <0.2) | | | | | | | | | | | | | |
