# Supplementary material for: Genetically predicted telomere length is associated with clonal somatic copy number alterations in peripheral leukocytes
Source: PLoS Genet. 2020 Oct 22;16(10):e1009078. doi: 10.1371/journal.pgen.1009078 (PMC7608979; doi:10.1371/journal.pgen.1009078)
Supplement: S6 Table — (DOCX) [file pgen.1009078.s009.docx]

| **S6 Table**. Mendelian randomization results using variants and summary statistics from Li et al. (2020) | | | | | |
| --- | --- | --- | --- | --- | --- |
|  | All Variants | |  | Pleiotropic Variants Removed^a^ | |
| Method | OR (95% CI) | p-value |  | OR (95% CI) | p-value |
| Maximum-likelihood | 1.79 (1.40-2.30) | 4.00x10^-6^ |  | 1.72 (1.48-2.00) | 7.51x10^-13^ |
| Simple median | 1.60 (1.30-1.98) | 9.05x10^-6^ |  | 1.62 (1.31-2.01) | 9.96x10^-6^ |
| Weighted median | 1.79 (1.45-2.21) | 7.44x10^-8^ |  | 1.70 (1.38-2.09) | 4.40x10^-7^ |
| IVW^b^ | 1.69 (1.33-2.15) | 1.75x10^-5^ |  | 1.69 (1.46-1.95) | 2.90x10^-12^ |
| MR-Egger | 3.35 (2.11-5.32) | 3.18x10^-7^ |  | 2.16 (1.40-3.32) | 4.79x10^-4^ |
| Intercept^c^ | 0.96 (0.94-0.99) | 0.0020 |  | 0.99 (0.96-1.01) | 0.2375 |
| ^a^Three variants (rs7705526, rs228595, rs34991172) were detected to have evidence of pleiotropy (FDR <0.2) | | | | | |
| ^b^Inverse-variance weighted  ^c^Intercept estimated from MR-Egger model | | | | | |
