# Supplementary material for: Genetically predicted telomere length is associated with clonal somatic copy number alterations in peripheral leukocytes
Source: PLoS Genet. 2020 Oct 22;16(10):e1009078. doi: 10.1371/journal.pgen.1009078 (PMC7608979; doi:10.1371/journal.pgen.1009078)
Supplement: S7 Table — (DOCX) [file pgen.1009078.s010.docx]

| **S7 Table**. Association between genetically-predicted telomere length and chromosome X SCNAs by event type and copy number change^a^ | | | | | | | |
| --- | --- | --- | --- | --- | --- | --- | --- |
|  | Univariable Model | |  | Multivariable Model | |  |  |
|  | OR (95% CI) | p-value |  | OR (95% CI) | p-value |  | p- value_het_^b^ |
| Overall | 1.01 (0.997-1.034) | 0.1118 |  | 1.04 (1.025-1.064) | 5.87x10^-6^ |  |  |
| Event Type |  |  |  |  |  |  | 0.7403 |
| Telomeric | 1.03 (0.982-1.076) | 0.2413 |  | 1.05 (1.000-1.097) | 0.0501 |  |  |
| Interstitial | 1.12 (0.886-1.419) | 0.3397 |  | 1.14 (0.902-1.449) | 0.2691 |  |  |
| Whole | 1.01 (0.992-1.032) | 0.2336 |  | 1.04 (1.022-1.064) | 5.91x10^-5^ |  |  |
| Copy Number Change |  |  |  |  |  |  | 0.0136 |
| Gain | 1.46 (1.164-1.840) | 0.0011 |  | 1.51 (1.196-1.904) | 5.29x10^-4^ |  |  |
| Loss | 1.01 (0.983-1.029) | 0.6094 |  | 1.03 (1.010-1.058) | 5.63x10^-3^ |  |  |
| Neutral | 1.05 (0.848-1.308) | 0.6400 |  | 1.07 (0.860-1.336) | 0.5376 |  |  |
| Undetermined | 1.02 (0.993-1.054) | 0.1387 |  | 1.05 (1.021-1.085) | 1.09x10^-3^ |  |  |
| Multivariable models control for age, age^2^, genetic ancestry, and detailed smoking status | | | | | |  |  |
| ^a^Only includes females |  |  |  |  |  |  |  |
| ^b^Denotes test of heterogeneity  age^2^= age-squared | |  |  |  |  |  |  |
