# Supplementary material for: Genetically predicted telomere length is associated with clonal somatic copy number alterations in peripheral leukocytes
Source: PLoS Genet. 2020 Oct 22;16(10):e1009078. doi: 10.1371/journal.pgen.1009078 (PMC7608979; doi:10.1371/journal.pgen.1009078)
Supplement: S8 Table — (DOCX) [file pgen.1009078.s011.docx]

| **S8 Table**. Association between genetically-predicted telomere length and chromosome Y loss by log_2_ R ratio^a^ | | | | | |
| --- | --- | --- | --- | --- | --- |
|  |  | Univariable Model | | Multivariable Model | |
| LRR | Cases (N) | OR (95% CI) | p-value | OR (95% CI) | p-value |
| Overall | 38,685 | 0.95 (0.939-0.960) | <2x10^-16^ | 0.97 (0.963-0.986) | 2.71x10^-5^ |
| -0.10 < LRR ≤ -0.05 | 7,417 | 0.96 (0.935-0.979) | 1.87x10^-4^ | 0.98 (0.957-1.004) | 0.1105 |
| -0.15 < LRR ≤ -0.10 | 2,728 | 0.94 (0.907-0.978) | 1.66x10^-3^ | 0.97 (0.929-1.004) | 0.0788 |
| -0.20 < LRR ≤ -0.15 | 1,196 | 0.99 (0.932-1.044) | 0.6286 | 1.10 (0.957-1.076) | 0.6175 |
| -0.25 < LRR ≤ -0.20 | 662 | 0.96 (0.892-1.038) | 0.3239 | 0.99 (0.916-1.071) | 0.8096 |
| -0.30 < LRR ≤ -0.25 | 392 | 0.99 (0.894-1.089) | 0.7912 | 1.01 (0.915-1.121) | 0.8029 |
| -0.35 < LRR ≤ -0.30 | 251 | 0.94 (0.830-1.062) | 0.3164 | 0.97 (0.855-1.100) | 0.6314 |
| -0.40 < LRR ≤ -0.35 | 196 | 0.89 (0.778-1.027) | 0.1133 | 0.91 (0.793-1.054) | 0.2152 |
| LRR ≤ -0.40 | 505 | 1.06 (0.970-1.155) | 0.2014 | 1.10 (1.007-1.206) | 0.0340 |
| Multivariable models control for age, age^2^, ethnicity, and detailed smoking status | | | | |  |
| ^a^Only includes males | |  |  |  |  |
| age^2^= age-squared |  |  |  |  |  |
